# Prioritizing FDA approved therapeutics for treating sepsis phenotypes: A network modeling approach based on neutrophil proteomics

**DOI:** 10.3389/fimmu.2025.1646141

**Published:** 2025-08-14

**Authors:** Jordan C. Langston, Dan Liu, Qingliang Yang, Salim Merali, Carmen Merali, Narender Singh, Jennifer L. Fisher, Balabhaskar Prabhakarpandian, Laurie E. Kilpatrick, Mohammad F. Kiani

**Affiliations:** ^1^ Department of Bioengineering, Temple University, Philadelphia, PA, United States; ^2^ Department of Mechanical Engineering, Temple University, Philadelphia, PA, United States; ^3^ School of Pharmacy, Temple University, Philadelphia, PA, United States; ^4^ Biotechnology, Energy, and Materials Division, CFD Research Corporation, Huntsville, AL, United States; ^5^ Center for Inflammation and Lung Research, Department of Microbiology, Immunology and Inflammation, Lewis Katz School of Medicine, Temple University, Philadelphia, PA, United States

**Keywords:** bioinformatics, drug repurposing, neutrophils, organ on chip, proteomics, sepsis

## Abstract

**Introduction:**

Sepsis is characterized by life-threatening organ dysfunction caused by dysregulated host response to infection. A key contributor is the disruption of neutrophil-endothelial interactions. Despite extensive research, there are no FDA-approved therapies that directly target altered neutrophil function in sepsis.

**Methods:**

We previously identified three functionally distinct neutrophil phenotypes in sepsis patients: Hyperimmune, Hypoimmune, and Hybrid, using clinical profiling, organ-on-chip models, and proteomics. In this study, we applied bioinformatics tools to elucidate the molecular pathways and druggable targets associated with each phenotype. Differentially expressed proteins were identified using ExpressAnalyst, while pathway enrichment and modeling were performed via Metascape and KEGG-based analyses. DrugBank and the Broad Institute Drug Repurposing Hub were queried to identify FDA-approved therapeutics. STRING and Cytoscape were used to build protein–protein interaction networks and prioritize hub targets.

**Results:**

In our study, the Hyperimmune and Hybrid neutrophil phenotypes had similar numbers of upregulated proteins, while the Hypoimmune and Hybrid neutrophil phenotypes had approximately the same numbers of downregulated proteins. Functional enrichment analysis highlighted several biological processes and pathways that impacted adhesion/migration patterns, such as calcium transport and neutrophil degranulation. Neutrophil pathway analysis highlighted nine differentially expressed proteins that were directly implicated in known neutrophil processes related to sepsis, such as leukocyte transendothelial migration. These findings were leveraged to identify FDA-approved therapeutics that could be repurposed to target proteins within each phenotype highlighting the impact in normalizing altered neutrophil-related responses such as adhesion, migration and pro-inflammatory mediator release. Finally, a protein-protein interaction network was employed to prioritize these target proteins within each phenotype using network analysis and identified three distinct drug targets across phenotypes that could modulate the neutrophil response in sepsis: VTN in the Hybrid phenotype, TRPV2 in the Hypoimmune phenotype and H2AC21 in the Hyperimmune phenotype.

**Discussion:**

Our integrative approach highlights phenotype-specific drug targets and FDA-approved candidates to modulate dysfunctional neutrophil responses in sepsis. This strategy supports a precision medicine framework for repurposing existing drugs based on neutrophil functional phenotyping.

## 1 Introduction

According to the Sepsis-3 definition, sepsis is characterized as life-threatening organ dysfunction resulting from a dysregulated host response to infection (1). Sepsis accounts for over 250,000 deaths annually in the US and is responsible for 20% of global mortality (2–4). In sepsis pathology, the dysregulation of the leukocyte adhesion cascade is a hallmark of disease progression. This results in increased neutrophil adhesion and migration across endothelial barriers into tissues, as well as increased barrier permeability, ultimately contributing to organ damage and enhanced mortality rates in sepsis patients (5, 6). At present, there are no FDA-approved therapeutics targeting the underlying pathophysiology of the disease (7), particularly the neutrophil-endothelial interactions. Additionally, approximately 150 potential druggable chemical entities that succeeded in treating sepsis in murine models have failed in clinical trials (7, 8) primarily as a result of the heterogeneous nature of sepsis—including sex, age, infection source, demographics, comorbidities, and importantly diversity in host response to infection and pathogen type, which can alter the clinical course of the disease (5). These differences, particularly in immune function and response to infection, limit the use of animal models for developing therapeutics for sepsis (7, 9, 10), as they inadequately mimic the various clinical manifestations of the human disease and differences in leukocyte composition between rodents and humans (7, 11). Thus, due to the of the lack of translation from bench-to-bedside, novel methods (e.g., *in silico* modeling and organ-on-chip (OoC) assays) are required to a) further our knowledge of the phenotypes of the disease leading to clinical presentation and precision medicine (12), b) develop novel testable hypotheses for the discovery of druggable candidates at a faster rate and a lower cost, and c) investigate how patient heterogeneity impacts the response to therapeutics in the disease (13).

Specifically, in sepsis, endothelial cells (ECs) are activated, resulting in enhanced neutrophil-EC interactions, disruption of EC barrier, upregulation of adhesion molecules and induction of apoptosis (14, 15). These events lead to increase neutrophil rolling, adhesion, migration across the barrier and excessive neutrophil trafficking into critical organs (e.g., lungs) and eventually, multiple organ dysfunction syndrome, if left uncontrolled. Furthermore, neutrophils can damage ECs through neutrophil extracellular trap formation (NETs), degranulation and release of reactive oxygen species, thus disrupting the EC glycocalyx and enhancing permeability through the breakdown of cell-cell junctions (12, 14, 16). Previously we used a synergistic combination of clinical and laboratory results from sepsis patients, a functional OoC assay and neutrophil proteomics to identify and validate three neutrophil functional phenotypes (i.e., Hyperimmune, Hypoimmune and Hybrid) in sepsis patients (16). *Ex vivo* neutrophils in the Hyperimmune phenotype exhibited increased adhesion and migration across the endothelium barrier in response to cytomix in our OoC; neutrophils in the Hypoimmune phenotype demonstrated blunted adhesion and migration patterns, while neutrophils in the Hybrid phenotype showed increased adhesion but blunted migration (16). These functional neutrophil phenotypes were associated with distinct proteomic signatures indicating significant intrinsic differences in protein expression among these neutrophil functional groups (16).

In this study, we propose a workflow to identify proteins targeting differentially expressed proteins (DEPs) unique to each functional neutrophil phenotype in sepsis patients, employing a synergistic approach that combines experimental proteomics and biological network modeling. Network modeling has been effective in discovering optimal drug targets in various contexts, including previously established targets for breast cancer (e.g., SRC proto-oncogene non-receptor tyrosine kinase (SRC), mechanistic target of rapamycin kinase (MTOR)) (17) and spinal cord injury (e.g., TNF, FOS, IL6) (18). We hypothesize that conducting functional enrichment analysis will reveal distinct biological processes associated with these neutrophil functional phenotypes while applying pathway analysis and protein-protein interaction (PPI) network analysis. This will offer an objective methodology for identifying phenotype-specific drug targets and examples of FDA-approved therapeutics that could potentially be repurposed for treatment of sepsis.

## 2 Materials and methods

### 2.1 Human neutrophil proteomic analysis

As previously reported (16), patients (ages 18–88 years old) in the Temple University Hospital Medical ICU who were diagnosed with sepsis or septic shock according to the Sepsis-3 definition (1) were eligible for enrollment in this study following written informed consent (Temple IRB protocol #24515), and a single 10-15cc of blood sample was obtained. Neutrophill isolation was started within 1 hour following the blood draw from sepsis patients employing standard isolation techniques such as ficoll-hypaque separation, dextran sedimentation, and hypotonic lysis to remove erythrocytes as previously described (16). Isolated neutrophils were not vortexed and were kept at room temperature to reduce the possibility of *ex vivo* neutrophil activation. The freshly isolated neutrophils were used in organ-on-chip experiments within an hour of isolation (16). Control samples were deidentified healthy adult donors through the Thrombosis Research Center Blood Program (Temple IRB protocol #24515) (16). For proteomic analysis, freshly isolated neutrophils were suspended in HBSS (2 x 10^6^ cells/ml), centrifuged and the cell pellets stored at -70°C prior to label-free global proteomic analysis (16). The proteomics analysis was performed for three neutrophil phenotypes as defined by their unique adhesion/migration patterns in our OoC as reported in a previous study (16). Samples were prepared and analyzed by mass spectrometry as described by our group previously (16). Mass spectra processing was performed with Proteome Discoverer version 2.5. The generated de-isotoped peak list was submitted to an in-house Mascot server 2.2.07 for searching against the Swiss-Prot database (Release 2013_01, version 56.6, 538,849 sequences), MSAmanda 2.0 database and Sequest HT database. Mascot, MS Amanda 2.0 and Sequest HT search parameters were set as follows: species, homo sapiens; enzyme, trypsin with maximal one missed cleavage; static modification, cysteine carbamidomethyl; 10 ppm mass tolerance for precursor peptide ions; 0.02 Da tolerance for MS/MS fragment ions. For dynamic modifications, oxidation/+15.995 Da (M) and N-terminal modification Met-loss/-131.040 Da (M) were used. Further bioinformatic analysis of the data was performed in R using RStudio (v.4.1.2). Pearson correlation coefficients (r) were calculated and transformed to Fisher z scale for a t-test with the Benjamini-Hochberg false discovery rate (FDR) algorithm to identify DEPs within neutrophil proteomes between phenotypes. Proteins with a fold change>2 and a FDR-adjusted p<0.01 were characterized as upregulated, while proteins with a fold change<0.5 and a FDR-adjusted p<0.01 were downregulated. DEPs were further characterized to identify those that are expressed in neutrophils.

### 2.2 Functional enrichment analysis

Metascape integrates protein annotation, membership search, interactome analysis and functional enrichment and uses over 40 independent databases within a single web service to provide a comprehensive analysis on omics data (19). Thus, this tool was used for functional enrichment analysis across the DEPs to obtain the enriched terms. Specifically, we use “terms” to indicate the pathways (e.g., KEGG) and biological processes (e.g., Gene Ontology) that are significant using a hypergeometric test/Fisher’s exact test (Benjamini-Hochberg adjusted-p value < 0.05). Terms were ranked by p-value [i.e., -log10(P value)]. Barcharts of enriched terms were created using the ggplot2 package in RStudio. ExpressAnalyst (20) was used to create UpSet plots of the upregulated and downregulated DEPs using the “Distinct” mode. Enriched terms were identified across druggable targets; however, due to the limited number of therapeutically targeted, uniquely expressed DEPs within each individual phenotype, pathway-level analyses were more informative when aggregating DEPs across phenotypes. Thus, we combined all the therapeutically targeted DEPs across phenotypes to show how DEPs associated with a given term may contribute to neutrophil function in a single phenotype or multiple phenotypes.

### 2.3 Identification of FDA-approved therapeutics and (pre) clinical trial therapeutics

The Harvard/MIT Broad Institute Drug Repurposing Hub (21) is an open-access, curated repository of >6000 compounds/therapeutics and >2000 targets; these compounds are in different preclinical and clinical phases of the drug development pipeline, including those that are approved and launched. Drugbank (version 6.0) is a repository that integrates drug data with drug target information; the database contains 4563 FDA-approved drugs, 6231 investigational drugs and 1,413,413 drug-drug interactions (22). In this study, we only included therapeutics from both databases that were categorized as “Launched” in the Drug Repurposing Hub or “Approved” from Drugbank since these therapeutics are already approved and could be potentially repurposed for sepsis treatment. “Launched” indicates a therapeutic that is FDA approved and is clinically available, whereas “Approved” indicates a therapeutic that is FDA approved that may or may not be clinically available. FDA-approved and (pre)clinical trial therapeutics from these two databases were used to target the DEPs within and across phenotypes in our study. Barcharts were created to illustrate which DEPs were targeted by therapeutics across phenotypes.

### 2.4 PPI network analysis

The Search Tool for the Retrieval of Interacting Genes/Proteins (STRING) database (version 12.0) (23) was used to create a protein-protein interaction (PPI) network of the DEPs that are targeted by FDA-approved therapeutics or pre (clinical) trial therapeutics. STRING creates PPI networks when there is evidence indicating a functional relationship between two proteins; proteins in these interactions can be functionally or indirectly associated. A full STRING network was created using all interaction sources (e.g., Textmining, Neighborhood, Experiments, Gene Fusion, Databases, Co-occurrence, Co-expression). The confidence level for the STRING network was set to low (0.15) to include the maximum number of proteins in the resulting PPIs (24). Disconnected nodes and protein names were hidden. The remaining parameters were default. The STRING network was then imported into Cytoscape (version 3.10.1) (25) for further network analysis and data visualization, and the cytoHubba app was used to identify and rank nodes in a PPI using the Degree parameter (26). Hubs are defined as the most connected proteins within the network that are responsible for sustaining network (degree-based circular PPIs) connectivity (27).

## 3 Results

### 3.1 Global proteomic analysis indicates significant differences in protein expression between phenotypes

**Figure 1:**
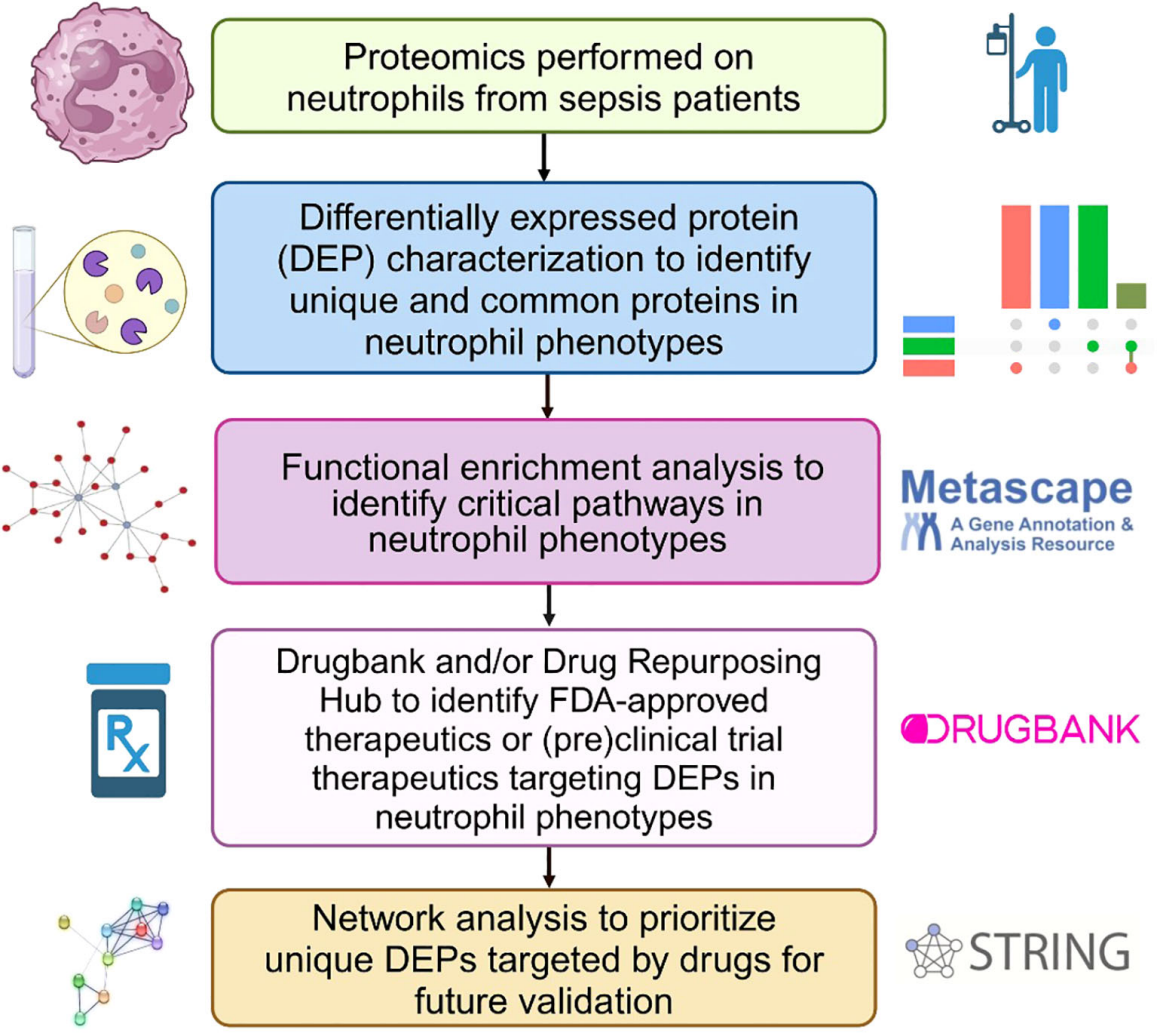
Bioinformatics workflow to identify and prioritize DEPs in neutrophil phenotypes that are targeted by FDA-approved therapeutics or classified as pre(clinical) trial therapeutics for sepsis treatment. This figure was created in BioRender.

**Figure 2:**
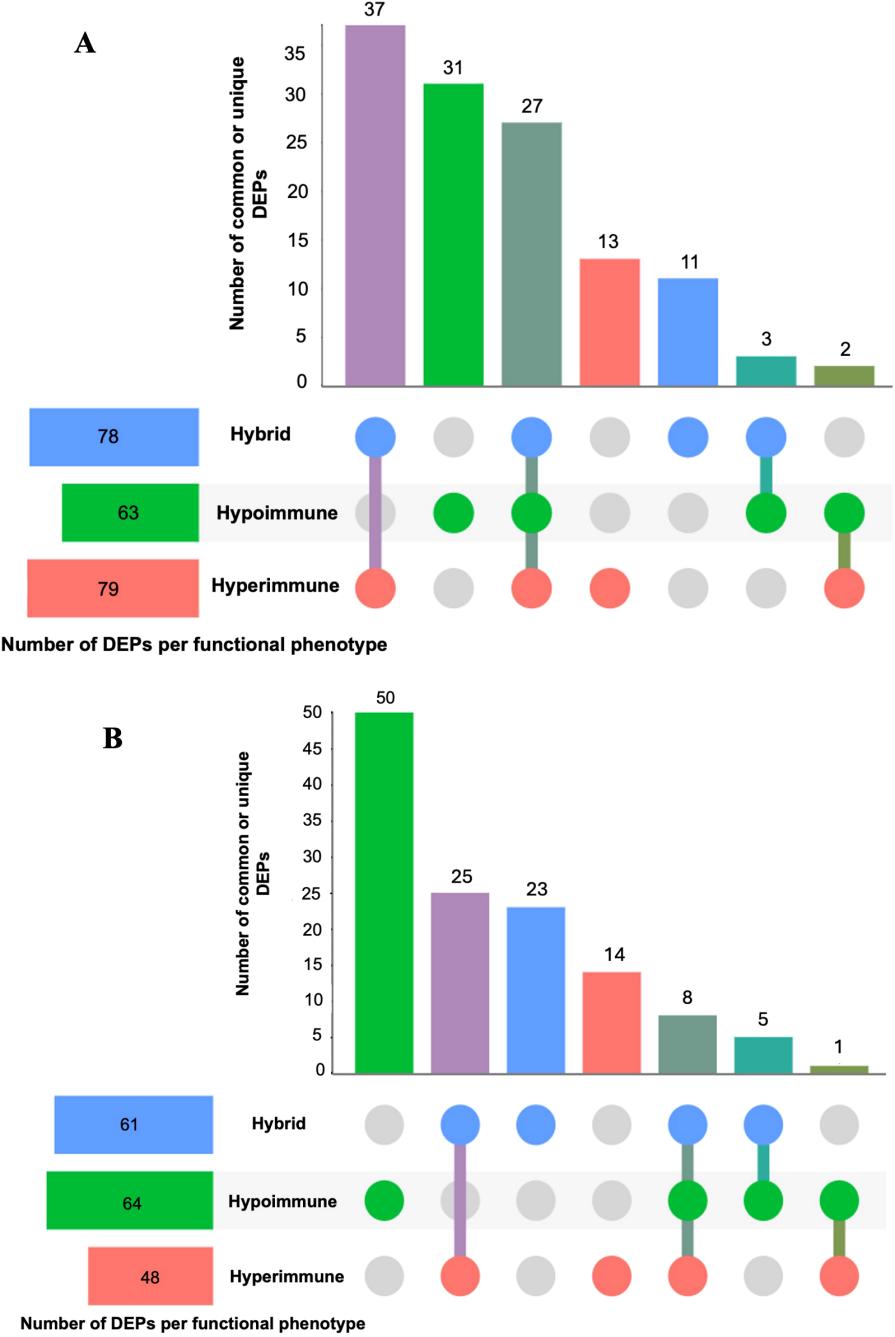
UpSet plots of neutrophil upregulated **(A)** and downregulated **(B)** differentially expressed proteins (DEPs) (compared to controls) within and between the three different functional neutrophil phenotypes. The horizontal bars show the total number of differentially expressed proteins identified in each phenotype, and the vertical bars (distinct size) indicate the number of DEPs that were unique or common across phenotypes. Dots that are connected indicate DEPs that are shared across phenotypes; while unconnected dots indicate DEPs that are unique within a phenotype. The purple vertical bar indicates the number of shared DEPs between the Hyperimmune and Hybrid phenotypes; the green bar indicates the number of DEPs unique to the Hypoimmune phenotype; the dark green bar indicates those DEPs that are common across all three phenotypes; the red bar indicates the number of DEPs unique to the Hypoimmune phenotype; the blue bar indicates the number of DEPs unique to the Hybrid phenotype; the turquoise bar indicates the number of shared DEPs between the Hybrid and Hypoimmune phenotypes and the forest green bar indicates the number of shared DEPs between the Hyperimmune and Hypoimmune phenotypes.

**Figure 1** shows the bioinformatics analysis workflow for this study. We use procurement analysis of three different neutrophils phenotypes in sepsis patients identified and validated previously (16) to not only perform functional enrichment analysis but also utilize network modeling (i.e., PPIs) to identify phenotype-specific drug targets and that could be used to repurpose FDA-approved and (pre)clinical trial therapeutics that target DEPs unique to each functional neutrophil phenotype. To test our hypothesis that functional enrichment analysis can identify terms (i.e. significant processes or pathways) associated with neutrophil phenotype DEPs, unbiased proteomic analysis of sepsis patient neutrophils was performed using mass spectrometry to discover the DEPs unique to each phenotype and common DEPs across phenotypes. **Figure 2** shows UpSet plots of the upregulated (**Figure 2A**) and downregulated (**Figure 2B**) DEPs. Hyperimmune and Hybrid phenotypes had approximately the same number of total upregulated proteins supporting the inference that the neutrophil proteomes in these patients were altered to a greater degree as compared to the Hypoimmune phenotype. Conversely, Hypoimmune and Hybrid phenotypes had the highest number of total downregulated proteins followed by the Hyperimmune phenotype further demonstrating differential regulation of protein expression in the different neutrophil phenotypes. Additionally, the Hypoimmune phenotype had the highest number of unique upregulated and downregulated proteins. These specific DEPs are listed in the “Common and unique upregulated and downregulated proteins in sepsis patient phenotypes” (**Supplementary Table 1**).

### 3.2 Functional enrichment analysis of protein targets identifies calcium signaling and cell division impacting neutrophil function

**Figure 3:**
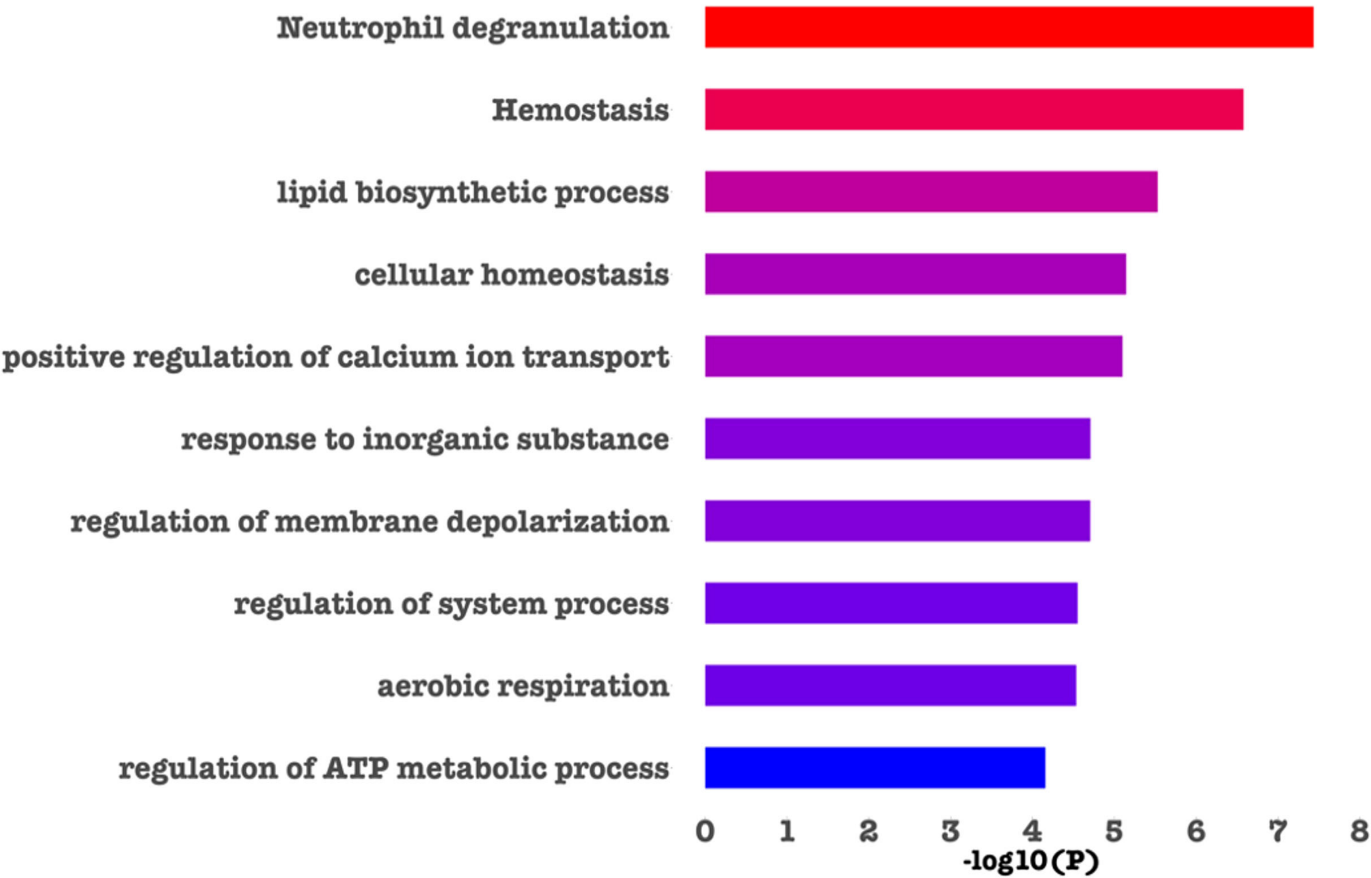
Barchart showing the top enriched categories in all the DEPs that are druggable by FDA-approved therapeutics. Terms are ranked based on decreasing statistical significance where the red bars indicate the most significant terms and the blue bars indicate the least significant terms. In this figure, and in agreement with the literature (30–32), we found that calcium signaling, cell division and neutrophil degranulation are critical processes impacting neutrophil adhesion/migration.

**Figure 4:**
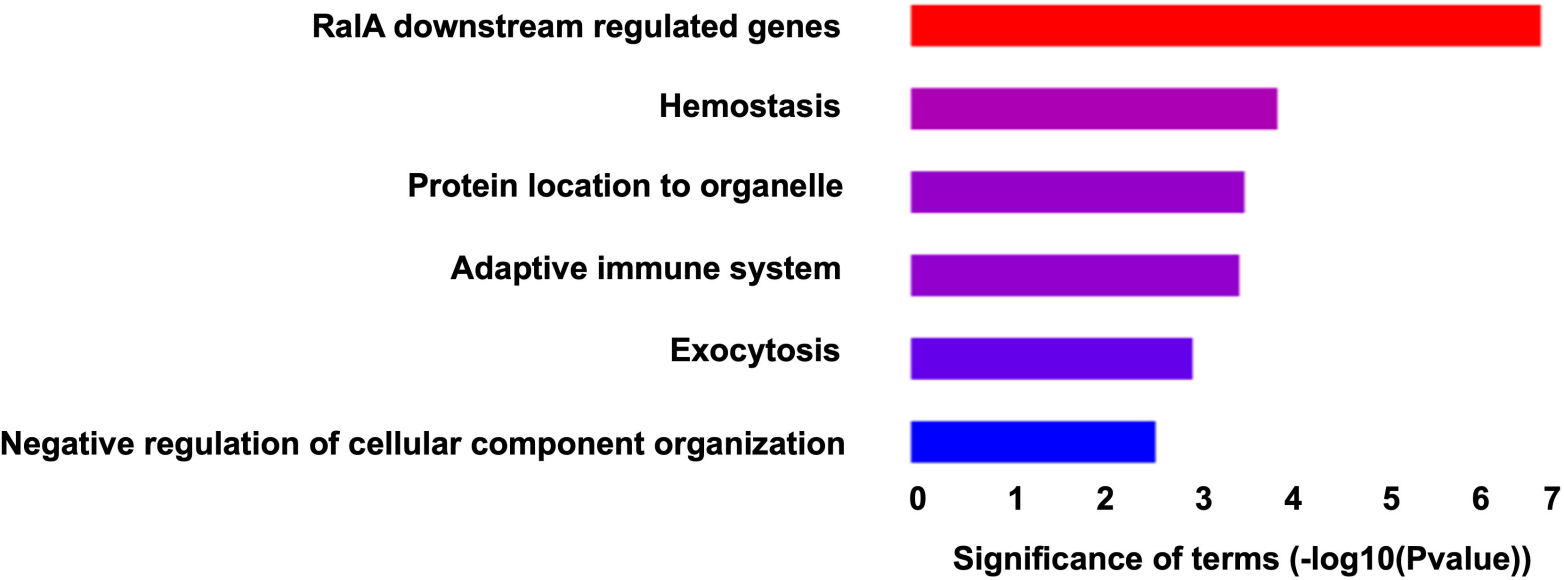
Barchart showing the top enriched categories in all the DEPs that are druggable by pre(clinical) trial therapeutics. Terms are ranked based on decreasing statistical significance where the red bars indicate the most significant terms and the blue bars indicate the least significant terms.

Metascape was used to identify those biological pathways and processes that were significantly enriched across the DEPs targeted by FDA-approved or (pre)clinical trial therapeutics, including terms that impact neutrophil adhesion/migration, as shown in **Figure 3** and **Figure 4** respectively. We performed functional enrichment analysis on the DEPs that are targeted by FDA-approved therapeutics and observed the association of the DEPs with calcium transport which suggests an increase in cytosolic calcium (28). Neutrophil rolling is not only mediated by selectins but also causes downstream release of endoplasmic reticulum calcium stores and leads to adhesion of neutrophils to the endothelium (29). For the enriched terms using the DEPs targeted by (pre)clinical trial therapeutics as input, Cell division control protein 42 (Cdc42 - a downstream gene of RAS Like Proto-Oncogene (RalA) signaling, **Figure 4**) is a critical regulator of cell polarity and in neutrophils, Cdc42, along with Wasp, controls neutrophil chemotaxis and transmigration to lung alveoli during inflammation (30). Proteins associated with each of these terms are listed in the “Metascape analysis of DEPs targeted by FDA-approved therapeutics and DEPs classified as pre(clinical) trial therapeutics” (**Supplementary Table 2**).

### 3.3 Neutrophil pathway analysis identifies drug targets across phenotypes impacting differential functional outcomes

**Figure 5:**
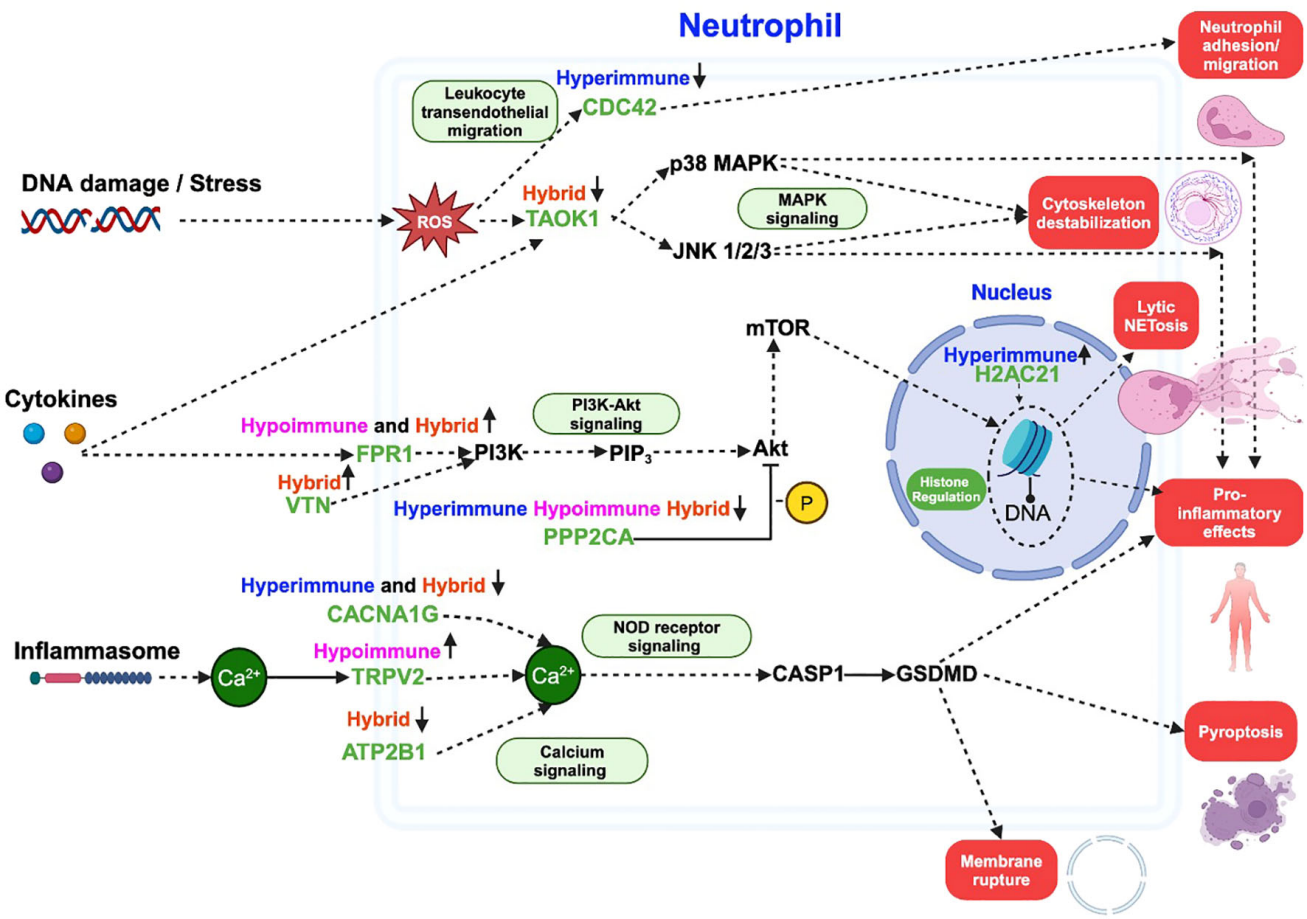
A schematic illustrating some of the critical pathways and processes involving differentially expressed neutrophil proteins (green) targeted by FDA-approved therapeutics. The other non-target protein pathways are shown in black, and downstream outcomes are highlighted in red blocks. The schematic is based on NETs formation pathway in KEGG, with additional pathways (green blocks) added to show the diverse roles of each protein across multiple signaling mechanisms leading to an outcome. Solid arrows (
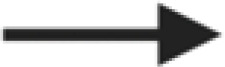
) indicate direct links between proteins, dashed arrows (
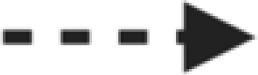
) represent indirect associations, and “T”-shaped solid arrows (
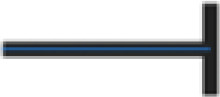
) indicate direct inhibition. Phenotypes are listed above each drug target and arrows next to each phenotype represent upregulation or downregulation. ROS refers to reactive oxygen species, -P indicates dephosphorylation and Ca^2+^ indicates calcium ion. This figure was created in BioRender.

Building on our central hypothesis that distinct biological processes and cellular pathways are differentially regulated in the three identified sepsis neutrophil functional phenotypes, we further identified specific drug targets within these pathways and their potential downstream effects on neutrophil-related outcomes such as adhesion, migration, neutrophil extracellular traps (NETs) formation, and pro-inflammatory mediator release. From the list of identified DEPs as potential drug targets for sepsis (listed in “All FDA-approved therapeutics and pre(clinical) trial therapeutics targeting the DEPs” **Supplementary Table 3**), nine DEPs (CDC42, TAOK1, FPR1, VTN, H2AC21, PPP2CA, CACNA1G, TRPV2, ATP2B1) were directly implicated in known neutrophil processes related to sepsis. As an example, these targets are illustrated in a simplified pathway model (created in Biorender) based on the KEGG NETs formation pathway as shown in **Figure 5**. Additionally, the identified drug targets were also mapped to the data collected from other pathways (e.g., MAPK signaling, PI3K-Akt signaling, NOD signaling, and calcium signaling events) in KEGG to highlight the diverse biological signaling pathways involved in neutrophil (patho)physiology. Some drug targets were specific to one phenotype, while others were common across multiple phenotypes, indicating their potential to mitigate neutrophil dysfunction in one or multiple phenotypes.

### 3.4 Unique identified FDA-approved therapeutics and (pre)clinical trial therapeutics targeting DEPs across sepsis phenotypes

**Figure 6:**
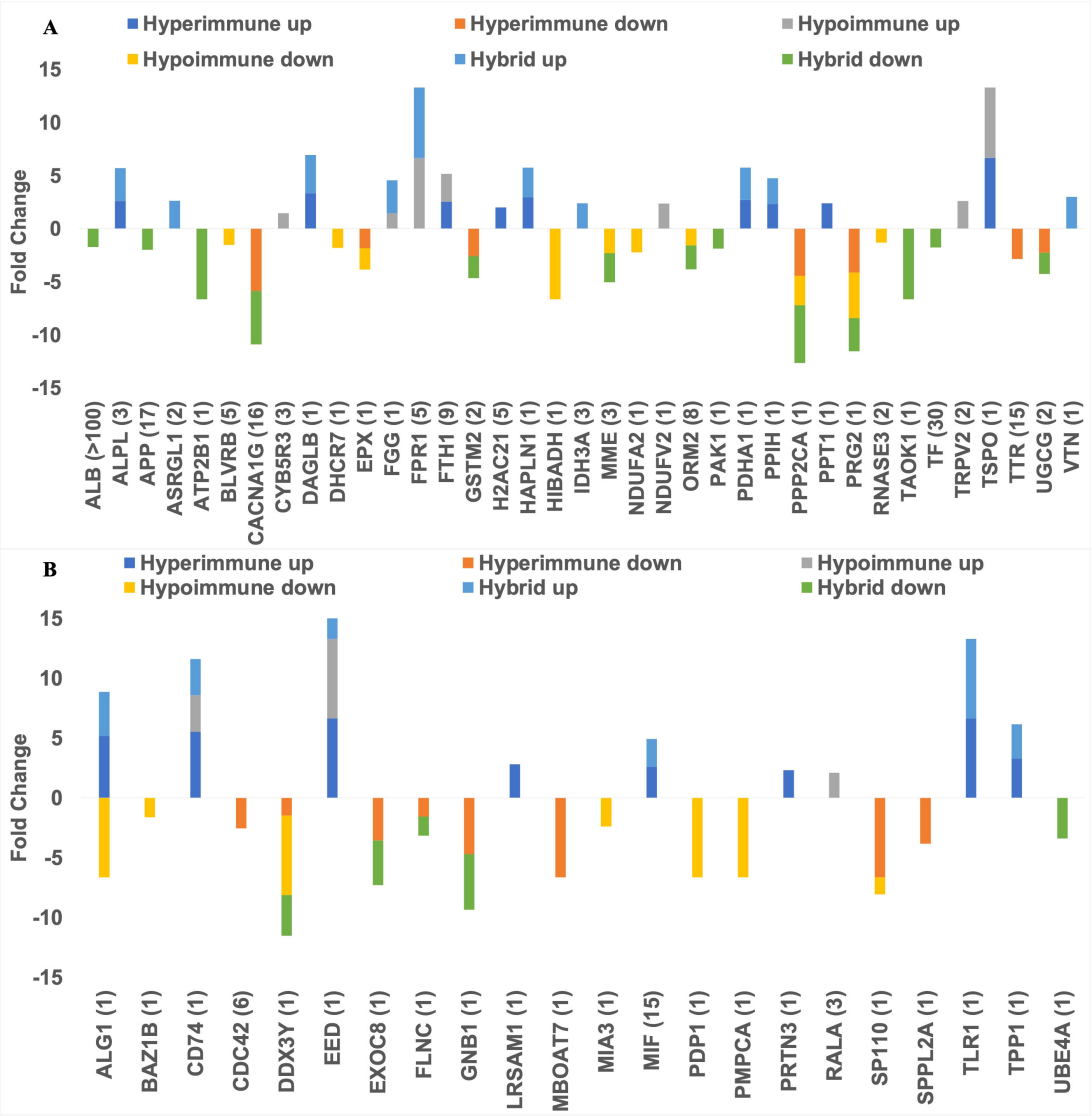
Barplots showing the number of FDA approved therapeutics targeting the upregulated and downregulated DEPs **(A)** and the number of (pre)clinical trial therapeutics targeting the DEPs **(B)**. The dark blue bar (top left) and orange bar (middle top) represents those DEPs that were uniquely upregulated or downregulated in the Hyperimmune phenotype respectively. The yellow bar (bottom left) and grey bar (top right) indicates those DEPs that were uniquely upregulated or downregulated in the Hypoimmune phenotype respectively. The light blue bar (middle bottom) and green bar (bottom right) shows those DEPs that were uniquely upregulated or downregulated in the Hybrid phenotype respectively.

**Figure 6** shows all the DEPs across phenotypes that are targeted by FDA-approved therapeutics (panel A) and those targeted by pre(clinical) trial therapeutics (panel B) plotted against their Fold Change as compared to healthy subjects. As shown in **Figure 6A**, nine upregulated DEPs are targeted by broad spectrum FDA-approved therapeutics (e.g., those therapeutics that target DEPs across more than one phenotype), while eight upregulated DEPs are targeted by FDA-approved therapeutics that are phenotype-specific (e.g., those drugs that target DEPs in one phenotype). All the approved and (pre)clinical trial therapeutics can be found in the “All FDA-approved therapeutics and pre(clinical) trial therapeutics targeting the DEPs” **Supplementary Table 3**. **Figure 6B** shows those DEPs that are targeted by pre(clinical) trial therapeutics. Even though these therapeutics are not approved, further experimentation can provide insight on what downstream effects they may have on underlying biological mechanisms and protein targets, prior to approval. The top DEP (i.e., the DEP with the most potential clinical candidates) was MIF (associated with the Hyperimmune and Hybrid phenotypes) for which 12 pre(clinical) trial therapeutics are in the pipeline. MIF has been shown to induce neutrophil migration *in vitro* (31). Several of these inhibitors such as caffeic-acid (32), ISO-1 (33), YZ9 (34) and 4-iodo-6-phenylpyrimidine (35) have been used in (pre)clinical sepsis studies; ISO-1, in particular, was found to inhibit leukocyte migration (36). Since these chemical therapeutics are not yet approved, there are no current indications. Even though we have identified those DEPs that are targeted by FDA-approved therapeutics or pre(clinical) therapeutics and play a role in neutrophil function and/or sepsis by investigating drug databases (e.g., Drugbank and/or Drug Repurposing Hub), we will use an objective approach (network analysis, see below) to prioritize these DEPs for each neutrophil phenotype for future sepsis treatment.

### 3.5 Network analysis identifies hubs targeted by FDA-approved therapeutics and (pre)clinical trial therapeutics

**Figure 7:**
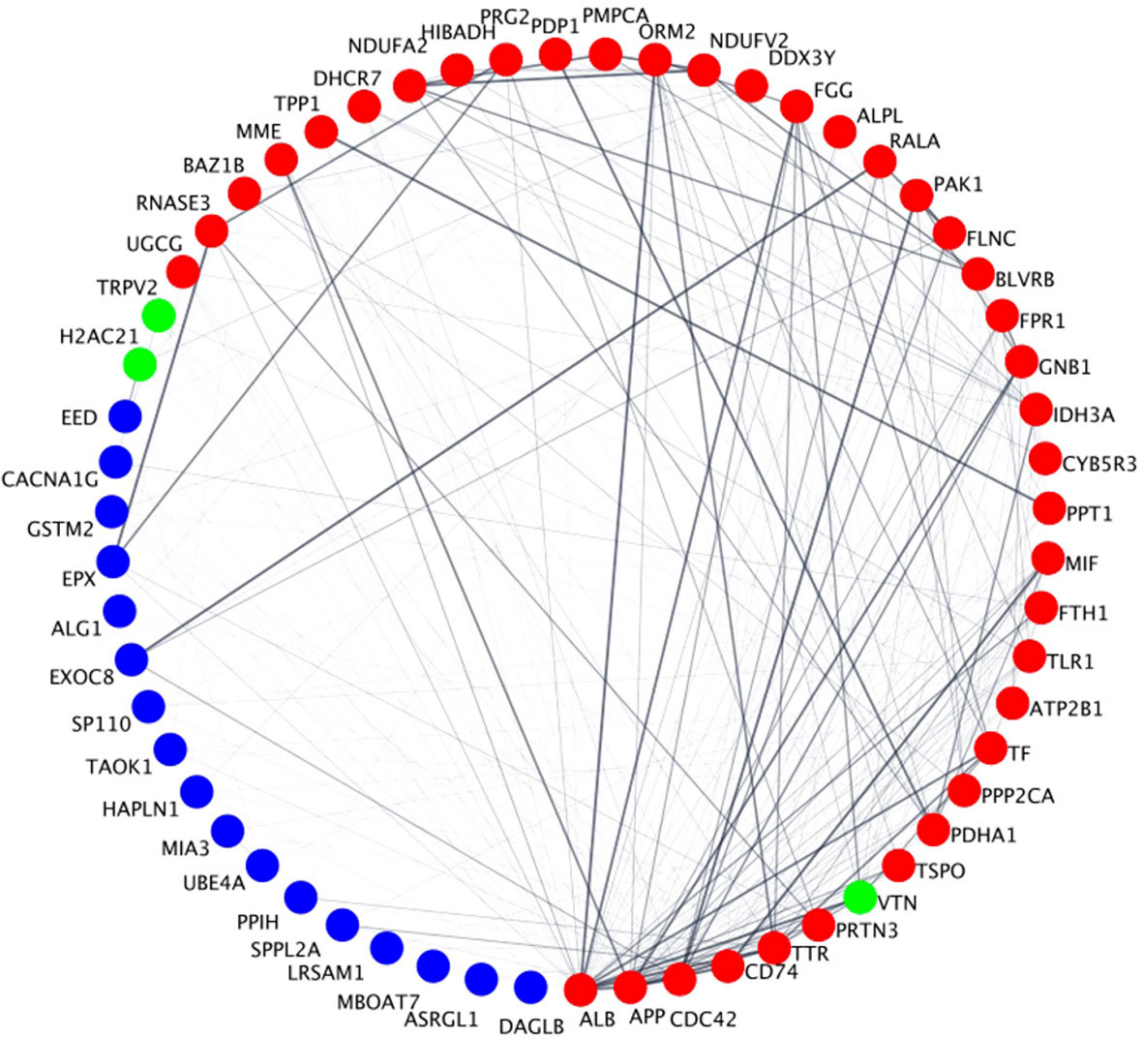
Degree-based circular STRING PPIs of the DEPs that are targeted by FDA-approved therapeutics and (pre)clinical trial therapeutics. Circles represent nodes (i.e., proteins) and edges between them represent physical or functional interactions. The thickness of the edge represents the amount of confidence associated with each interaction; thicker edges have greater data support (from the literature, experiments, etc.) associated with it compared to thinner edges. Node size is proportional to the degree of the proteins and is presented in a clockwise fashion (e.g., ALB has the highest degree and DAGLB has the lowest degree). Red nodes are classified as hubs, blue nodes are non-hubs and green nodes are unique upregulated hubs that play a role in the NETs pathway in **Figure 5** in a specific neutrophil phenotype. Specifically, VTN is unique in the Hybrid phenotype, TRPV2 in the Hypoimmune phenotype and H2AC21 in the Hyperimmune phenotype.

To prioritize the identified unique DEPs that are targeted by FDA-approved therapeutics for the potential treatment of sepsis, we deployed a network biology approach centered around identifying key hubs within biological networks. This methodology has been effective in uncovering optimal drug targets in various contexts, including previously established targets for breast cancer (e.g., SRC, MTOR) (17) and spinal cord injury (e.g., TNF, FOS, IL6) (18). By integrating network biology with protein expression data, we prioritize DEPs that are unique to these immune cells and use the PPI network utilizing STRING database to identify clustered hubs within these networks. **Figure 7** shows a STRING network of all the DEPs that are targeted by FDA-approved therapeutics and (pre)clinical trial therapeutics. Network analysis in **Figure 5** identified hubs as indicated in red (hubs), green (unique upregulated hubs in a phenotype), or blue (non-hubs). Specifically, the unique hub target of currently FDA-approved therapeutics was H2AC21 (Hyperimmune, degree=6), VTN (Hybrid, degree=15) and TRPV2 (Hypoimmune, degree=5). **Table 1** specifically shows examples of FDA-approved therapeutics (small molecules) targeting the unique upregulated DEPs from **Figure 6** and highlights additional information on these hub targets. Thus, networks analysis indicates that targets of currently FDA-approved therapeutics have high degree (number of connections to other nodes) and should be prioritized for treating each phenotype. Network statistics for the network in **Figure 7** as well as the results from the CytoHubba ranking analysis are included in the “STRING network analysis of FDA-approved therapeutics and (pre)clinical trial targeting neutrophil DEPs” file in the **Supplementary Table 4**.

**Table 1:** List of FDA-approved therapeutics that specifically target the unique upregulated differentially expressed proteins obtained using STRING network analysis in Cytoscape.

Functional phenotype(s)	Degree (number of connections with other proteins)	Drug(s)/small molecule(s)	DEP(s) and (Fold change in protein expression as compared to control)	DEP(s)’ inflammatory/neutrophil pathway(s) of interest	Reference citing drug in neutrophil and/or sepsis study
Hyperimmune	6	Polysialic acid, Heparin, C-reactive protein, Tirofiban, Thrombomodulin/activated protein C	H2AC21 (3)	Neutrophil extracellular trap formation: Histone/DNA packaging	(39)
Hypoimmune	5	Amiloride	TRPV2 (6)	NOD-like receptor signaling: Non-selective cation channel	(42, 45, 46)
Hybrid	15	RGDfv	VTN (11)	PI3K-Akt signaling: Glycoprotein	(47)

Only those therapeutics that are inhibitors of their selected targets are listed in this Table. Other therapeutics from our study are listed in “All FDA-approved therapeutics and pre(clinical) trial therapeutics targeting the DEPs (**Supplementary Table 3**).

All the FDA-approved therapeutics (small molecules) and their targets and pathways presented in **Table 1** have been shown to play a role in sepsis and/or neutrophil functions (see Discussion).

## 4 Discussion

The repurposing of FDA-approved therapeutics for diseases or indications outside their originally approved intent is an emerging field of research with significant potential to reduce the transition time of a therapeutic from bench to bedside, as well as to lower production costs and attrition percentages (37, 38). In this study, we used a bioinformatic, network biology approach to identify and repurpose FDA-approved therapeutics that could potentially be repurposed to treat sepsis. We identified DEPs within and across the three phenotypes (Hyperimmune, Hypoimmune, and Hybrid) and characterized the ontological and pathway roles of all the druggable DEPs across phenotype, demonstrating how DEPs associated with a given term may contribute to neutrophil function in a single phenotype or multiple phenotypes. Furthermore, we developed a neutrophil-specific pathway model highlighting the contribution of unique and/or common druggable DEPs to neutrophil downstream processes and elucidating other pathways that these therapeutics could target. We constructed a PPI network and used network analysis to discover the top-ranked, unique, druggable hubs across the phenotypes, and then mapped these hubs to their corresponding targets. Thus, our study is one of the first to incorporate proteomics, bioinformatics, *in vitro* and clinical data in the identification of FDA-approved potential therapeutics that could be repurposed to treat sepsis.

Relevance of several of the targets identified in this study to neutrophil function have been reported in the literature. For example, polysialic acid binds to histones *in vitro* using a single chain variable fragment antibody approach, reduces histone and NET cytotoxicity and indirectly decreases neutrophil adhesion; thus, it could mitigate sepsis damage (39, 40) in the Hyperimmune phenotype by binding to H2AC21. Since patients in the Hyperimmune phenotype had worse clinical outcomes compared to the Hypoimmune and Hybrid phenotypes and a proteomic signature consisting of more detrimental neutrophil patterns (16), the molecular effect of this therapeutic on the Hyperimmune’s neutrophil adhesion/migration patterns can be potentially beneficial to this group of patients. In addition, C-reactive protein reduces histone-mediated toxicity *in vitro* and in animal models and prevents calcium influx which can lead to downstream neutrophil consequences (39, 41). Another potential therapeutic identified in this study, Amiloride, suppresses the inflammatory response (42, 43), endothelial cell activation, chemokine production (42) and neutrophil migration (44), making it a candidate for Transient receptor potential cation channel subfamily V member 2 (TRPV2) (45, 46) in the Hypoimmune phenotype. Lastly, the RGD-blocking peptide binds to its RGD motif to reduce VTN’s antiapoptotic effects, leading to reduced neutrophil adhesion/migration patterns (47). Thus, this therapeutic could be repurposed to target VTN in the Hybrid phenotype. Patients in the Hybrid phenotype had enhanced adhesion but blunted migration, yet when activated, neutrophils in this phenotype could potentially accumulate in the vasculature (16), and thus a therapeutic that could mitigate this effect needs to be validated.

There are several possible limitations in this study. Even though this is the first study of its kind which utilizes ICU patients in a single medical center to identify phenotype-specific drug targets and FDA-approved candidates with potential to modulate dysfunctional neutrophil responses in sepsis, we only studied ICU patients with advanced sepsis. This highlights the fact that our model has not been tested in patients with early or developing sepsis such as a patient population that would be encountered in the emergency department. Additional studies may be required to determine if the omic profile of sepsis patients changes significantly during the progression of the disease. Validation of potential therapeutics identified in this study in *in vitro*, *in vivo* and/or *in silico* models is necessary to ensure they have a significant effect in mitigating neutrophil adhesion/migration damage in sepsis patients and potentially lead to better clinical outcomes. These potential therapeutics can be validated using the microphysiological system incorporated in our previous study (16). We have previously shown that a novel therapeutic for treating sepsis (PKCδ TAT inhibitory peptide) similarly reduces neutrophil migration and adhesion in both our microphysiological system using primary human cells and in an animal model of sepsis (14, 15). In addition, the identification of hubs in the PPI network indicates which nodes play a crucial role in regulating multiple processes/pathways within and across phenotypes and thus should be further investigated as druggable candidates. The PPI network was constructed using the STRING database. However, additional edges and proteins may be documented in the literature but not curated and included in this database, rendering the network incomplete. Therefore, detailed curation of the literature to incorporate novel edges and proteins into the network in the future could further the predictive power of these networks. Furthermore, the incorporation of multi-omic (48–52) datasets and creating network models that contain a variety of biological entitles (e.g., proteins, transcription factors, genes, metabolites etc.) would provide a more complete picture of the underlying biological processes in septic neutrophils and identify even more protein targets for potential therapeutics.

In summary, functional enrichment analysis of DEPs from different neutrophil phenotypes generated sepsis-related pathways and processes can advance our understanding of the underlying molecular mechanisms of this heterogeneous disease. Network analysis has been used to uncover drug targets (53–55) and for drug repurposing leading to novel clinical applications of approved drugs (56, 57). In this study, we used a novel application of network analysis leveraging human neutrophil DEPs in sepsis to identify drug targets, an approach that has not been explored when compared to traditional methods like gene profiling or RNA sequencing (58–60). Focusing on neutrophil functional phenotypes allows us to prioritize DEPs that are unique to these immune cells, potentially revealing targeted therapeutic strategies that could enhance precision treatment options for sepsis based on specific phenotypes. Furthermore, we used the PPI network using STRING database to identify clustered hubs within these networks to not only shed light on the underlying biological mechanisms of sepsis but also enhance our ability to predict and prioritize potential therapeutic targets. This is of paramount importance since there are currently no FDA-approved therapeutics that modulate and restore the immune response in sepsis. Although a few studies have investigated FDA-approved therapeutics repurposing for sepsis (38, 61), to our knowledge, this is the first therapeutic repurposing study that leverages a combination of clinical, functional phenotyping and proteomics of sepsis patient neutrophils tools to identify examples of FDA-approved therapeutics that can be repurposed for treating sepsis. Since these phenotypes have been validated by showing that they correlate with disease severity (16), this study provides a roadmap for achieving precision medicine in sepsis to identify the right therapeutic for the right patient at the right time.

## Data Availability

The datasets presented in this study can be found in online repositories. The names of the repository/repositories and accession number(s) can be found below: https://www.ebi.ac.uk/pride/archive/, PXD041007.

## Glossary

ALB: Albumin
ALG1: ALG1 chitobiosyldiphosphodolichol beta-mannosyltransferase
ALPL: Alkaline phosphatase
APP: Amyloid beta precursor protein
ASRGL1: Asparaginase and isoaspartyl
ATP2B1: ATPase plasma membrane Ca2+ transporting 1
BAZ1B: Bromodomain adjacent to zinc finger domain 1B
BLVRB: Biliverdin reductase B
BP: Biological process
CACNA1G: Calcium voltage-gated channel subunit alpha1G
CD2: Cluster of differentiation 2
CD247: Cluster of differentiation 247
CD74: Cluster of differentiation 74
Cdc42: Cell division cycle 42
COVID 19: Coronavirus disease 2019
CYB5R3: Cytochrome b5 reductase 3
DAGLB: Diacylglycerol lipase beta
DDX3Y: DEAD-box helicase 3 Y-linked
DHCR7: 7-dehydrocholesterol reductase
DEPs: Differentially expressed proteins
EED: Embryonic ectoderm development
EPX: Eosinophil peroxidase
EXOC8: Exocyst complex component 8
FDA: Food and Drug Administration
FDR: False discovery rate
FGG: Fibrinogen gamma chain
FLNC: Filamin C
FOS: Fos proto-oncogene, AP-1 transcription factor subunit
FPR1: Formyl peptide receptor 1
FTH1: Ferritin heavy chain 1
GNB1: G protein subunit beta 1
GO: Gene ontology
GSTM2: Glutathione S-transferase mu 2
H2AC21: H2A clustered histone 21
HAPLN1: Hyaluronan and proteoglycan link protein 1
HIBADH: 3-hydroxyisobutyrate dehydrogenase
ICU: Intensive care unit
IDH3A: Isocitrate dehydrogenase (NAD(+)) 3 catalytic subunit alpha
IL6: Interleukin 6
KEGG: Kyoto Encyclopedia of Genes and Genomes
LRSAM1: Leucine rich repeat and sterile alpha motif containing 1
KLBR1: Killer cell lectin like receptor B1
MAPK: Mitogen-activated protein kinase
MBOAT7: Membrane bound O-acytransferase domain containing 7
MIA3: MIA SH3 domain ER export factor 3
MIF: Macrophage migration inhibitory factor
MME: Membrane metalloendopeptidase
MTOR: Mechanistic target of rapamycin kinase
NDUFA2: NADH:ubiquinone oxidoreductase subunit A2
NDUFV2: NADH:ubiquinone oxidoreductase core subunit V2
NETs: Neutrophil extracellular traps
NOD: Nucleotide-binding oligomerization domain-like receptors
OoC: Organ-on-chip
ORM2: Orosomucoid 2
PAK1: p21 (RAC1) activated kinase 1
PDHA1: Pyruvate dehydrogenase E1 subunit alpha 1
PDP1: Pyruvate dehydrogenase phosphatase catalytic subunit 1
PI3k-Akt: Phosphoinositide 3-kinase-Protein kinase B
PMPCA: Peptidase, mitochondrial processing subunit alpha
PPIs: Protein-protein interaction networks
PPP2CA: Protein phosphatase 2 catalytic subunit alpha
PPIH: Peptidylprolyl isomerase H
PPT1: Palmitoyl-protein thioesterase 1
PRG2: Proteoglycan 2, pro eosinophil major basic protein
RALA: RAS like proto-oncogene
RNASE3: Ribonuclease A family member 3
SRC: SRC proto-oncogene, non-receptor tyrosine kinase
SP110: SP110 nuclear body protein
SPPL2A: Signal peptide peptidase like 2A
STRING: Search Tool for the Retrieval of Interacting Genes/Proteins
TAOK1: TAO kinase 1
TF: Transferrin
TLR1: Toll-like receptor 1
TNF: Tumor necrosis factor
TRPV2: Transient receptor potention cation channel subfamily V member 2
TPP1: Tripeptidyl peptidase 1
TSPO: Translocator protein
TTR: Transthyretin
UBE4A: Ubiquitination factor E4A
UGCG: UDP-glucose ceramide glucosyltransferase
VTN: Vitronectin
Wasp: WASP actin nucleation promoting factor.
