# Supplementary material for: Prioritizing FDA approved therapeutics for treating sepsis phenotypes: A network modeling approach based on neutrophil proteomics
Source: Front Immunol. 2025 Aug 14;16:1646141. doi: 10.3389/fimmu.2025.1646141 (PMC12391923; doi:10.3389/fimmu.2025.1646141)
Supplement: Supplementary file 1 [file Table1.docx]

**Hyperimmune unique upregulated proteins**

| Symbol | Fold Change | P value |
| --- | --- | --- |
| NCAPH | 100 | 2.1409E-16 |
| SNX15 | 100 | 2.1409E-16 |
| JAGN1 | 49.305 | 1.1373E-08 |
| TBC1D24 | 16.601 | 0.00103318 |
| MRPL12 | 16.231 | 5.4883E-06 |
| DPY19L3 | 13.428 | 0.00090154 |
| LRSAM1 | 7.018 | 0.00252856 |
| TSTD1 | 6.11 | 0.00052968 |
| PPT1 | 5.253 | 0.00240237 |
| PRTN3 | 4.979 | 0.0003868 |
| CAB39L | 4.703 | 0.00767968 |
| H2AC21 | 3.84 | 0.00859534 |
| GNAI2 | 3.769 | 0.00757246 |

**Hypoimmune unique upregulated proteins**

| Symbol | Fold Change | P value |
| --- | --- | --- |
| AMDHD2 | 4.502 | 0.00354853 |
| CARD8 | 2.712 | 0.0081064 |
| CDK11B | 2.32 | 0.00045075 |
| CSNK2B | 3.278 | 1.3566E-06 |
| CTTN | 4.577 | 0.00764329 |
| CYB5R3 | 2.736 | 1.8763E-05 |
| DNPH1 | 3.188 | 0.00270158 |
| EIF3K | 9.012 | 5.3472E-09 |
| EIF4G3 | 12.723 | 2.236E-06 |
| EMB | 2.106 | 0.00048015 |
| LRP10 | 2.756 | 0.00012046 |
| MBNL1 | 2.509 | 0.00045075 |
| MLKL | 2.003 | 0.00933704 |
| MMRN1 | 2.3 | 0.00475044 |
| NDUFV2 | 5.171 | 0.00385926 |
| PLS3 | 5.983 | 6.4825E-05 |
| PPP1R3D | 3.499 | 8.2917E-05 |
| RALA | 4.242 | 0.00455745 |
| RBM3 | 9.325 | 1.0935E-07 |
| RBMXL1 | 4.321 | 1.3893E-08 |
| SELP | 3.249 | 0.0001841 |
| SF3B6 | 2.286 | 0.00474058 |
| SLC3A2 | 7.319 | 0.00032565 |
| SNRPA | 3.042 | 0.00711404 |
| SPARC | 4.794 | 2.4125E-12 |
| TANGO2 | 4.099 | 2.4332E-05 |
| THUMPD1 | 2.802 | 0.00014451 |
| TM9SF2 | 2.965 | 0.0057437 |
| TRPV2 | 6.038 | 0.00162126 |
| UQCRQ | 5.008 | 0.00181878 |
| VIPAS39 | 3.381 | 0.00166117 |

**Hybrid unique upregulated proteins**

| Symbol | Fold Change | P value |
| --- | --- | --- |
| ADGRE2 | 7.214 | 1.1021E-05 |
| ASRGL1 | 6.193 | 0.00328166 |
| FOLR3 | 21.262 | 3.9092E-13 |
| GGH | 4.231 | 0.00995989 |
| IDH3A | 5.217 | 0.00045312 |
| MYH11 | 100 | 2.1641E-16 |
| PIN1 | 4.932 | 0.00056587 |
| RPL31 | 5.765 | 0.00760674 |
| RPS20 | 4.991 | 0.00055185 |
| VTN | 11.405 | 6.2518E-05 |
| WNT7A | 6.761 | 0.00012112 |

**Hyperimmune-Hypoimmune upregulated proteins**

| Symbol | Hyperimmune Fold Change | Hypoimmune Fold Change | Hyperimmune P value | Hypoimmune P value |
| --- | --- | --- | --- | --- |
| NINJ1 | 100 | 27.928 | 2.14089E-16 | 3.45031E-11 |

**Hyperimmune-Hybrid upregulated proteins**

| Symbol | Hyperimmune Fold Change | Hybrid Fold Change | Hyperimmune P value | Hybrid P value |
| --- | --- | --- | --- | --- |
| ABRACL | 4.416 | 4.268 | 0.001479798 | 0.00827771 |
| ALG1 | 35.68 | 12.89 | 1.18181E-06 | 0.00386652 |
| ALPL | 6.036 | 8.575 | 0.000505961 | 1.0866E-06 |
| C16orf54 | 100 | 100 | 2.14089E-16 | 2.1641E-16 |
| C7orf25 | 5.494 | 4.238 | 0.00086751 | 0.00554631 |
| CEACAM1 | 100 | 100 | 2.14089E-16 | 2.1641E-16 |
| CST7 | 15.814 | 22.624 | 0.00123374 | 0.00031366 |
| CUTA | 7.715 | 8.217 | 2.3994E-05 | 1.0919E-06 |
| DAGLB | 10.031 | 12.177 | 0.000444726 | 5.7916E-05 |
| GNG5 | 5.956 | 7.383 | 0.000338232 | 1.9389E-06 |
| HAPLN1 | 7.836 | 6.872 | 3.7531E-05 | 3.7787E-05 |
| IFITM1 | 100 | 100 | 2.14089E-16 | 2.1641E-16 |
| IFRD1 | 100 | 100 | 2.14089E-16 | 2.1641E-16 |
| INTS5 | 100 | 100 | 2.14089E-16 | 2.1641E-16 |
| MIF | 6.003 | 5.02 | 3.42971E-05 | 0.00124846 |
| MYADM | 100 | 100 | 2.14089E-16 | 2.1641E-16 |
| NPL | 100 | 100 | 2.14089E-16 | 2.1641E-16 |
| PDHA1 | 6.567 | 8.171 | 0.005107119 | 0.00019 |
| PIP4K2C | 100 | 100 | 2.14089E-16 | 2.1641E-16 |
| PPIH | 4.961 | 5.382 | 0.006382082 | 0.00334965 |
| RAB33A | 100 | 100 | 2.14089E-16 | 2.1641E-16 |
| RPL34 | 9.355 | 11.197 | 2.40841E-06 | 3.3075E-09 |
| RPL9 | 9.974 | 6.376 | 1.47813E-06 | 0.00064972 |
| RPS29 | 4.569 | 8.221 | 0.007679684 | 3.8444E-06 |
| SDF2L1 | 100 | 100 | 2.14089E-16 | 2.1641E-16 |
| SEH1L | 5.519 | 4.576 | 0.000838675 | 0.00315149 |
| SH3BGRL3 | 23.003 | 17.47 | 1.76596E-12 | 2.5696E-11 |
| SMARCD2 | 36.73 | 11.403 | 1.52376E-06 | 0.00802721 |
| SRSF10 | 4.926 | 4.392 | 0.00194489 | 0.0028157 |
| SUMO2 | 100 | 100 | 2.14089E-16 | 2.1641E-16 |
| TBC1D8 | 100 | 100 | 2.14089E-16 | 2.1641E-16 |
| TLR1 | 100 | 100 | 2.14089E-16 | 2.1641E-16 |
| TPP1 | 9.641 | 7.347 | 2.17558E-08 | 1.3987E-05 |
| TPT1 | 7.309 | 5.528 | 1.9936E-06 | 0.00042787 |
| TRIOBP | 17.731 | 13.984 | 0.000771728 | 0.00657483 |
| TUBA1C | 100 | 100 | 2.14089E-16 | 2.1641E-16 |
| USP25 | 100 | 100 | 2.14089E-16 | 2.1641E-16 |

**Hypoimmune-Hybrid upregulated proteins**

| Symbol | Hypoimmune Fold Change | Hybrid Fold Change | Hypoimmune P value | Hybrid P value |
| --- | --- | --- | --- | --- |
| FGG | 2.739 | 8.516 | 2.0793E-06 | 1.8306E-06 |
| FPR1 | 100 | 100 | 2.41878E-16 | 2.1641E-16 |

**Common upregulated proteins**

| Symbol | Hyperimmune Fold Change | Hypoimmune Fold Change | Hybrid Fold Change | Hyperimmune P value | Hypoimmune P value | Hybrid P value |
| --- | --- | --- | --- | --- | --- | --- |
| AAK1 | 100 | 100 | 100 | 2.14089E-16 | 2.41878E-16 | 2.1641E-16 |
| ABCB7 | 100 | 100 | 100 | 2.14089E-16 | 2.41878E-16 | 2.1641E-16 |
| BCL7C | 100 | 100 | 100 | 2.14089E-16 | 2.41878E-16 | 2.1641E-16 |
| CD74 | 45.603 | 8.357 | 8.111 | 2.14089E-16 | 7.85675E-14 | 6.2226E-05 |
| CPD | 100 | 100 | 100 | 2.14089E-16 | 2.41878E-16 | 2.1641E-16 |
| EED | 100 | 100 | 100 | 2.14089E-16 | 2.41878E-16 | 2.1641E-16 |
| FTH1 | 5.898 | 2.109 | 6.146 | 4.36552E-05 | 3.88863E-05 | 0.00013007 |
| IFI30 | 100 | 100 | 100 | 2.14089E-16 | 2.41878E-16 | 2.1641E-16 |
| INTS1 | 100 | 100 | 100 | 2.14089E-16 | 2.41878E-16 | 2.1641E-16 |
| ITGB3 | 4.026 | 2.452 | 4.837 | 0.007302651 | 8.008E-05 | 0.00357919 |
| LANCL2 | 100 | 100 | 100 | 2.14089E-16 | 2.41878E-16 | 2.1641E-16 |
| MTX2 | 100 | 100 | 100 | 2.14089E-16 | 2.41878E-16 | 2.1641E-16 |
| NCAPG | 100 | 100 | 100 | 2.14089E-16 | 2.41878E-16 | 2.1641E-16 |
| PABPN1 | 26.828 | 24.357 | 29.07 | 1.81474E-11 | 2.41878E-16 | 2.496E-11 |
| PTP4A2 | 100 | 100 | 100 | 2.14089E-16 | 2.41878E-16 | 2.1641E-16 |
| RTCA | 11.706 | 10.563 | 11.152 | 1.32337E-06 | 2.41878E-16 | 1.7194E-05 |
| SDR39U1 | 100 | 100 | 100 | 2.14089E-16 | 2.41878E-16 | 2.1641E-16 |
| SLC35A5 | 17.487 | 5.425 | 20.098 | 0.000183935 | 0.003859255 | 0.00011461 |
| SLC39A11 | 100 | 100 | 100 | 2.14089E-16 | 2.41878E-16 | 2.1641E-16 |
| SNX14 | 100 | 100 | 100 | 2.14089E-16 | 2.41878E-16 | 2.1641E-16 |
| SRSF6 | 100 | 100 | 100 | 2.14089E-16 | 2.41878E-16 | 2.1641E-16 |
| SSH3 | 100 | 100 | 100 | 2.14089E-16 | 2.41878E-16 | 2.1641E-16 |
| THAP11 | 100 | 100 | 100 | 2.14089E-16 | 2.41878E-16 | 2.1641E-16 |
| TOR2A | 100 | 100 | 100 | 2.14089E-16 | 2.41878E-16 | 2.1641E-16 |
| TSEN34 | 100 | 100 | 100 | 2.14089E-16 | 2.41878E-16 | 2.1641E-16 |
| UBL5 | 100 | 100 | 100 | 2.14089E-16 | 2.41878E-16 | 2.1641E-16 |
| XPOT | 100 | 100 | 100 | 2.14089E-16 | 2.41878E-16 | 2.1641E-16 |

**Hyperimmune unique downregulated proteins**

| Symbol | Fold Change | P value |
| --- | --- | --- |
| ANTXR2 | 0.306 | 0.00171361 |
| CDC42 | 0.17 | 0.00016765 |
| CRNKL1 | 0.01 | 2.1409E-16 |
| CYB561D2 | 0.123 | 0.00220928 |
| EPX | 0.281 | 0.00067071 |
| H2BC13 | 0.01 | 2.1409E-16 |
| MBOAT7 | 0.01 | 2.1409E-16 |
| OLAH | 0.01 | 2.1409E-16 |
| RETN | 0.024 | 2.176E-11 |
| RWDD1 | 0.343 | 0.00557752 |
| SIGLEC14 | 0.182 | 0.00740384 |
| SP110 | 0.01 | 2.1409E-16 |
| SPPL2A | 0.07 | 8.4297E-05 |
| TTR | 0.137 | 7.2999E-06 |
| WDR11 | 0.169 | 0.00015307 |

**Hypoimmune unique downregulated proteins**

| Symbol | Fold Change | P value |
| --- | --- | --- |
| AASDHPPT | 0.07 | 2.9314E-06 |
| ALG1 | 0.01 | 2.4188E-16 |
| BAZ1B | 0.325 | 3.6563E-06 |
| BLVRB | 0.346 | 1.9602E-05 |
| CBX1 | 0.351 | 0.00480262 |
| CERS2 | 0.01 | 2.4188E-16 |
| CLC | 0.34 | 1.2686E-05 |
| CLEC12A | 0.432 | 0.00515739 |
| DENR | 0.346 | 0.00629858 |
| DHCR7 | 0.282 | 0.00969741 |
| DHX36 | 0.01 | 2.4188E-16 |
| DNAJC1 | 0.01 | 2.4188E-16 |
| EIF2B3 | 0.01 | 2.4188E-16 |
| EML3 | 0.01 | 2.4188E-16 |
| ERGIC1 | 0.01 | 2.4188E-16 |
| EXOC6 | 0.01 | 2.4188E-16 |
| GDAP2 | 0.279 | 0.00246995 |
| GHITM | 0.01 | 2.4188E-16 |
| HDDC2 | 0.01 | 2.4188E-16 |
| HIBADH | 0.01 | 2.4188E-16 |
| HMGA1 | 0.159 | 2.6124E-07 |
| IFIT2 | 0.281 | 0.00068638 |
| ISG15 | 0.157 | 5.7866E-05 |
| JAML | 0.01 | 2.4188E-16 |
| MIA3 | 0.191 | 0.00373962 |
| MRPS17 | 0.01 | 2.4188E-16 |
| MX1 | 0.44 | 0.00702879 |
| NDUFA2 | 0.212 | 0.00439702 |
| NIPBL | 0.01 | 2.4188E-16 |
| PAF1 | 0.01 | 2.4188E-16 |
| PCNP | 0.01 | 2.4188E-16 |
| PDP1 | 0.01 | 2.4188E-16 |
| PITPNM1 | 0.167 | 0.00029735 |
| PMPCA | 0.01 | 2.4188E-16 |
| PRG3 | 0.116 | 1.6597E-13 |
| PRPF38B | 0.01 | 2.4188E-16 |
| RNASE3 | 0.398 | 0.00057242 |
| RUSF1 | 0.01 | 2.4188E-16 |
| SEC13 | 0.341 | 0.00429209 |
| SH2D3C | 0.01 | 2.4188E-16 |
| SIGLEC9 | 0.01 | 2.4188E-16 |
| SMARCD2 | 0.01 | 2.4188E-16 |
| SSH2 | 0.01 | 2.4188E-16 |
| TBC1D23 | 0.01 | 2.4188E-16 |
| TNFAIP6 | 0.01 | 2.4188E-16 |
| TNFAIP8 | 0.376 | 0.00201564 |
| VPS36 | 0.356 | 8.2879E-05 |
| WDFY4 | 0.17 | 2.4188E-16 |
| WTAP | 0.371 | 0.00424265 |
| XPO6 | 0.01 | 2.4188E-16 |

**Hybrid unique downregulated proteins**

| Symbol | Fold Change | P value |
| --- | --- | --- |
| ALB | 0.301 | 0.0044248 |
| ANP32B | 0.275 | 0.00176636 |
| APOA1 | 0.201 | 0.00827771 |
| APOA2 | 0.197 | 0.00944156 |
| APOB | 0.098 | 0.00198768 |
| APP | 0.252 | 0.00077137 |
| ATP2B1 | 0.01 | 2.1641E-16 |
| CD101 | 0.176 | 0.00456054 |
| DDX3Y | 0.095 | 0.00456054 |
| ECE1 | 0.01 | 2.1641E-16 |
| FLNC | 0.327 | 0.00958986 |
| GIGYF2 | 0.01 | 2.1641E-16 |
| IL6R | 0.01 | 2.1641E-16 |
| LETM1 | 0.256 | 0.00111611 |
| MAVS | 0.01 | 2.1641E-16 |
| MME | 0.151 | 6.5876E-06 |
| ORM2 | 0.212 | 0.00918546 |
| OXR1 | 0.062 | 5.9416E-05 |
| PAK1 | 0.273 | 0.00209026 |
| PPP1R12C | 0.116 | 0.00940949 |
| SEC62 | 0.129 | 0.00737254 |
| SULT1A1 | 0.313 | 0.00486826 |
| TAOK1 | 0.01 | 2.1641E-16 |
| TBC1D24 | 0.01 | 2.1641E-16 |
| TF | 0.293 | 0.00327373 |
| TMEM120A | 0.125 | 0.0068911 |
| UBE4A | 0.095 | 0.00140748 |
| VIRMA | 0.01 | 2.1641E-16 |

**Hyperimmune-Hybrid downregulated proteins**

| Symbol | Hyperimmune Fold Change | Hybrid Fold Change | Hyperimmune P value | Hybrid P value |
| --- | --- | --- | --- | --- |
| ATG16L2 | 0.01 | 0.01 | 2.14089E-16 | 2.1641E-16 |
| BCL10 | 0.01 | 0.01 | 2.14089E-16 | 2.1641E-16 |
| CACNA1G | 0.017 | 0.031 | 2.14089E-16 | 2.1641E-16 |
| CAMP | 0.311 | 0.285 | 0.002022737 | 0.00260313 |
| CAP2 | 0.144 | 0.149 | 4.35718E-08 | 9.7239E-07 |
| CD200R1 | 0.069 | 0.126 | 0.00013292 | 0.00958986 |
| COPS8 | 0.266 | 0.275 | 0.007864744 | 0.00877457 |
| CRISP3 | 0.198 | 0.198 | 0.001097457 | 6.134E-05 |
| CRYL1 | 0.108 | 0.196 | 1.52838E-07 | 5.7012E-05 |
| DEFA1 | 0.148 | 0.194 | 7.26383E-08 | 3.5306E-05 |
| EXOC8 | 0.085 | 0.075 | 1.35001E-09 | 5.7016E-12 |
| GNB1 | 0.038 | 0.04 | 4.73269E-15 | 9.5252E-15 |
| GSTM2 | 0.167 | 0.238 | 5.15371E-07 | 0.00100204 |
| H1-4 | 0.194 | 0.165 | 0.000437776 | 0.00021031 |
| IFIT3 | 0.211 | 0.226 | 5.65898E-05 | 0.00011795 |
| IGSF6 | 0.099 | 0.122 | 3.71572E-11 | 4.3145E-08 |
| LACTB2 | 0.111 | 0.093 | 1.177E-06 | 1.1174E-08 |
| LST1 | 0.144 | 0.028 | 1.60107E-05 | 2.1641E-16 |
| NDE1 | 0.087 | 0.069 | 0.001538092 | 0.00069633 |
| PDCD4 | 0.206 | 0.231 | 0.002429232 | 0.00177406 |
| PLP2 | 0.159 | 0.115 | 7.28322E-05 | 2.9453E-08 |
| PRG2 | 0.057 | 0.116 | 2.14089E-16 | 2.0411E-08 |
| RASSF3 | 0.148 | 0.249 | 7.26383E-08 | 0.0006438 |
| RBL2 | 0.263 | 0.191 | 0.005452713 | 0.00035155 |
| RPS5 | 0.091 | 0.086 | 6.34954E-07 | 8.151E-06 |
| SSR4 | 0.048 | 0.046 | 2.14089E-16 | 2.1641E-16 |
| SVIP | 0.193 | 0.221 | 0.000620495 | 0.00039402 |
| SYMPK | 0.01 | 0.01 | 2.14089E-16 | 2.1641E-16 |
| TGOLN2 | 0.095 | 0.11 | 0.001895764 | 0.00698455 |
| TMBIM1 | 0.01 | 0.01 | 2.14089E-16 | 2.1641E-16 |
| UBE2L3 | 0.242 | 0.206 | 0.000110029 | 7.141E-05 |
| UGCG | 0.208 | 0.249 | 0.001365009 | 0.00387365 |

**Hyperimmune-Hypoimmune downregulated proteins**

| Symbol | Hyperimmune Fold Change | Hypoimmune Fold Change | Hyper P value | Hypoimmune P value |
| --- | --- | --- | --- | --- |
| EPX | 0.281 | 0.245 | 0.0006707 | 4.52905E-10 |

**Hypoimmune-Hybrid downregulated proteins**

| Symbol | Hypoimmune Fold Change | Hybrid Fold Change | Hypoimmune P value | Hybrid P value |
| --- | --- | --- | --- | --- |
| ORM2 | 0.333 | 0.212 | 0.004976709 | 0.00918546 |
| OXR1 | 0.097 | 0.062 | 2.82293E-05 | 5.9416E-05 |
| SULT1A1 | 0.344 | 0.313 | 1.67923E-05 | 0.00486826 |
| MME | 0.201 | 0.151 | 1.45004E-08 | 6.5876E-06 |
| DDX3Y | 0.01 | 0.095 | 2.41878E-16 | 0.00456054 |

**Common downregulated proteins**

| Symbol | Hyperimmune Fold Change | Hypoimmune Fold Change | Hybrid Fold Change | Hyperimmune P value | Hypoimmune P value | Hybrid P value |
| --- | --- | --- | --- | --- | --- | --- |
| LST1 | 0.144 | 0.35 | 0.028 | 1.6011E-05 | 0.00065641 | 2.1641E-16 |
| IFIT3 | 0.211 | 0.379 | 0.226 | 5.659E-05 | 0.00372651 | 0.00011795 |
| TGOLN2 | 0.095 | 0.063 | 0.11 | 0.00189576 | 1.114E-08 | 0.00698455 |
| BCL10 | 0.01 | 0.01 | 0.01 | 2.1409E-16 | 2.4188E-16 | 2.1641E-16 |
| PRG2 | 0.057 | 0.051 | 0.116 | 2.1409E-16 | 2.4188E-16 | 2.0411E-08 |
| PPP2CA | 0.046 | 0.147 | 0.023 | 1.7476E-11 | 2.3014E-09 | 3.7111E-13 |
| SVIP | 0.193 | 0.276 | 0.221 | 0.0006205 | 9.0109E-06 | 0.00039402 |
| CD200R1 | 0.069 | 0.201 | 0.126 | 0.00013292 | 0.0011385 | 0.00958986 |
