# Supplementary material for: Prioritizing FDA approved therapeutics for treating sepsis phenotypes: A network modeling approach based on neutrophil proteomics
Source: Front Immunol. 2025 Aug 14;16:1646141. doi: 10.3389/fimmu.2025.1646141 (PMC12391923; doi:10.3389/fimmu.2025.1646141)
Supplement: Supplementary file 2 [file Table2.docx]

**Metascape enrichment analysis of differential expressed proteins targeted by FDA-approved therapeutics**

| **GroupID** | **Category** | **Description** | **LogP** | **Log(q-value)** | **Symbols** |
| --- | --- | --- | --- | --- | --- |
| Summary | Reactome Gene Sets | Neutrophil degranulation | -7.4345461 | -3.0900163 | CYB5R3  FPR1  FTH1  MME  ORM2  PRG2  RNASE3  EPX  ALPL  TSPO  TF  ATP2B1  APP |
| Summary | Reactome Gene Sets | Hemostasis | -6.5793814 | -2.7885754 | ALB  APP  ATP2B1  FGG  ORM2  PPP2CA  TF  DAGLB  FPR1  FTH1 |
| Summary | GO Biological Processes | lipid biosynthetic process | -5.5298909 | -1.9635123 | TSPO  DHCR7  CYB5R3  GSTM2  PPT1  UGCG  DAGLB  APP  IDH3A  ALPL |
| Summary | GO Biological Processes | cellular homeostasis | -5.1452422 | -1.756165 | ALPL  APP  ATP2B1  FTH1  PPT1  TF  TAOK1  CACNA1G  TRPV2  ALB |
| Summary | GO Biological Processes | positive regulation of calcium ion transport | -5.1006948 | -1.756165 | ATP2B1  TSPO  GSTM2  TRPV2  TF  HAPLN1  MME  ALPL  UGCG |
| Summary | GO Biological Processes | response to inorganic substance | -4.7078157 | -1.5651178 | ALPL  TSPO  FGG  PPP2CA  TF  CACNA1G |
| Summary | GO Biological Processes | regulation of membrane depolarization | -4.7055276 | -1.5651178 | ALB  TSPO  CACNA1G  DHCR7  PPT1  UGCG  APP  MME  FGG |
| Summary | GO Biological Processes | regulation of system process | -4.5505571 | -1.444333 | APP  ATP2B1  TSPO  FGG  GSTM2  CACNA1G  DAGLB  CYB5R3  MME  TRPV2 |
| Summary | GO Biological Processes | aerobic respiration | -4.5335903 | -1.444333 | IDH3A  NDUFA2  NDUFV2  PDHA1  PPT1  UGCG  DAGLB  GSTM2 |
| Summary | GO Biological Processes | regulation of ATP metabolic process | -4.157768 | -1.2111782 | APP  TSPO  PPP2CA  FGG  ORM2  FPR1  GSTM2  HAPLN1  DAGLB  TAOK1 |

**Metascape enrichment analysis of differential expressed proteins targeted by (pre)clinical trial therapeutics**

| **GroupID** | **Category** | **Description** | **LogP** | **Log(q-value)** | **Symbols** |
| --- | --- | --- | --- | --- | --- |
| Summary | WikiPathways | RalA downstream regulated genes | -7.1377284 | -2.7942607 | CDC42  RALA  EXOC8  MIF  PRTN3  LRSAM1  GNB1  MIA3  FLNC |
| Summary | Reactome Gene Sets | Hemostasis | -4.1533244 | -0.588008 | CD74  CDC42  GNB1  MIF  PRTN3  MIA3  TLR1  MBOAT7  RALA |
| Summary | GO Biological Processes | protein localization to organelle | -3.7795558 | -0.2811862 | TPP1  RALA  PMPCA  LRSAM1  MIA3 |
| Summary | Reactome Gene Sets | Adaptive Immune System | -3.7207943 | -0.2804166 | CD74  CDC42  TLR1  UBE4A  LRSAM1  MIF |
| Summary | GO Biological Processes | exocytosis | -3.1908539 | -0.0778352 | RALA  EXOC8  MIA3  CDC42 |
| Summary | GO Biological Processes | negative regulation of cellular component organization | -2.7703951 | 0 | CDC42  PRTN3  BAZ1B  LRSAM1  CD74 |
