# Supplementary material for: Prioritizing FDA approved therapeutics for treating sepsis phenotypes: A network modeling approach based on neutrophil proteomics
Source: Front Immunol. 2025 Aug 14;16:1646141. doi: 10.3389/fimmu.2025.1646141 (PMC12391923; doi:10.3389/fimmu.2025.1646141)
Supplement: Supplementary file 3 [file Table3.docx]

| **Functional phenotype(s)** | **Drug(s)/small molecule(s)** | **DEP(s) and (fold change in protein expression as compared to control)**  **FDA-approved drugs targeting differential expressed proteins and studied in neutrophil and/or sepsis studies** | **DEP(s) inflammatory/neutrophil pathway(s) of interest** | **Mechanism of action(s)** | **Reference citing drug in sepsis and/or neutrophil study** |
| --- | --- | --- | --- | --- | --- |
| (Hyperimmune, Hybrid) | Amifostine | ALPL (6.0, 8.5) | AGE-RAGE pathway (elicits MAPK and NFKB signaling) | Reducing agent | Batchik et al., 2022 |
|  |  |  |  |  |  |
| (Hyperimmune, Hybrid) | Pyridoxine | ALPL (6.0, 8.5) | AGE-RAGE pathway (elicits MAPK and NFKB signaling) | Vitamin B6 agonist | Deng et al., 2013 |
|  |  |  |  |  |  |
| Hybrid | L-aspartic acid | ASRGL1 (6.1) | Metabolic and amino acid metabolism pathway | Metallic radical formation agonist | Su et al., 2015 |
|  |  |  |  |  |  |
| (Hyperimmune, Hybrid) | L-proline | PPIH (4.9, 5.3) | Metabolism of RNA; Viral infection | Glutamate receptor agonist | Nunes et al., 2023 |
|  |  |  |  |  |  |
| Hyperimmune | Palmitic acid | PPT1 (5.2) | Fatty acid elongation/metabolism pathway | Enzyme antagonist | Peng et al., 2021 |
|  |  |  |  |  |  |
| (Hypoimmune, Hybrid, Hypoimmune, (Hyperimmune, Hybrid), Hypoimmune, Hypoimmune, Hypoimmune) | NADH | (CYB5R3: 2.7; IDH3A: 5.2, NDUFV2: 5.1, PDHA1: (6.5, 8.1), BLVRB .34, DHCR7: .28, HIBADH: .01, NDUFA2: .21) | CYB5R3: Amino sugar and nucleotide sugar metabolism, Neutrophil degranulation | Vitamin B12 agonist | Ye et al., 2022 |
|  |  |  | IDH3A: TCA and respiratory electron transport pathway |  |  |
|  |  |  | NDUFV2: Oxidative phosphorylation |  |  |
|  |  |  | PDHA1: HIF-1 signaling, Glycolysis, TCA cycle |  |  |
|  |  |  | BLVRB: Cell response to stress, Riboflavin metabolism |  |  |
|  |  |  | DHCR7: Cholesterol biosynthesis |  |  |
|  |  |  | HIBADH: Metabolism of amino acids and derivatives |  |  |
|  |  |  | NDUFA2: TCA and respiratory electron transport chain |  |  |
|  |  |  |  |  |  |
| Hypoimmune | Cannabidiol | TRPV2 (6.0) | NOD-like receptor signaling; Inflammatory mediator regulation of TRP channels | Agonist (Cannabinoid receptors) | Maayah et al., 2022 |
|  |  |  |  |  | McHugh et al., 2008 |
| Hypoimmune | Flavin adenine dinucleotide (FAD) | CYB5R3 (2.7) | CYB5R3: Amino sugar and nucleotide sugar metabolism, Neutrophil degranulation | Cofactor, Vitamin B2 agonist | Protti et al., 2006 |
| (Hypoimmune, Hybrid, Hybrid, Hybrid, Hyperimmune) | Copper | (CYB5R3: 2.7, IDH3A: 5.2, TF: .29, APP: .25, TTR: .13) | CYB5R3: Amino sugar and nucleotide sugar metabolism, Neutrophil degranulation | Antioxidant; Cofactor | Yang et al., 2022 |
|  |  |  | IDH3A: TCA and respiratory electron transport pathway |  | Lominadze et al., 2004 |
|  |  |  | APP: Cytokine signaling in immune system, Post-translational protein phosphorylation, TLR cascades, Signaling by Interleukins |  |  |
|  |  |  | TTR: Neutrophil degranulation, Metabolism of vitamins |  |  |
|  |  |  |  |  |  |
| (Hypoimmune, Hybrid) | Thrombin alfa Human Thrombin/Thrombin/Alteplase | FGG (2.7, 8.5) | Complement and coagulation; NETs; Integrin cell surface signaling | Agonist/Binder | Hosac et al., 2002 |
|  |  |  |  |  | Moser et al., 1990 |
| (Hyperimmune, Hypoimmune, Hybrid) | Iron | FTH1 (5.8, 2.1, 6.1) | FTH1: Ferroptosis, Necroptosis, Neutrophil degranulation | Agonist of hemoglobin | Xia et al., 2016 |
|  |  |  | TF: HIF-1 signaling, Ferroptosis, Post-translational protein phosphorylation |  |  |
| Hybrid | Manganese | IDH3A (5.2) | IDH3A: TCA and respiratory electron transport pathway | Antioxidant | Nin et al., 2004 |
|  |  |  | TF: HIF-1 signaling, Ferroptosis, Post-translational protein phosphorylation |  | Neeli et al, 2023 |
| Hypoimmune | Amiloride | TRPV2 (6.0) | NOD-like receptor signaling, Inflammatory mediator regulation of TRP channels | Sodium channel antagonist | Sikes et al., 2005 |
| (Hyperimmune, Hybrid) | Hyaluronic acid | HAPLN1 (7.8, 6.8) | Extracellular matrix organization/proteoglycans; Cell adhesion | Binder | Lee et al., 2020 |
|  |  |  |  |  | Forrester et al., 1982 |
| Hypoimmune | Riboflavin 5 phosphate sodium/Flavin mononucleotide | BLVRB (.34) | Cell response to stress; Riboflavin metabolism | Vitamin B agonist; iron agonist | Toyosawa et al., 2004 |
|  |  |  |  |  |  |
| Hypoimmune | Methylene Blue | BLVRB (.34) | Cell response to (chemical) stress/stimuli; Riboflavin metabolism | Guanylyl cyclase antagonist; Nitric oxide production antagonist | Ibarra-Estrada et al., 2023 |
|  |  |  |  |  | Hong et al., 1998 |
| (Hyperimmune, Hypoimmune) | Melatonin | EPX (.28, .24) | Neutrophil degranulation | Melatonin receptor agonist; nitric oxide synthase inhibitor | Liu et al., 2022 |
|  |  |  |  |  | Sahna et al., 2008 |
| (Hypoimmune, Hybrid) | Sacubitril | MME (.20, .15) | Neutrophil degranulation; Metabolism of angiotensinogen to angiotensins | Neprilysin inhibitor | Wang et al., 2023 |
|  |  |  |  |  |  |
| (Hypoimmune, Hybrid) | Chlorpromazine | ORM2 (.33, .21) | Neutrophil degranulation; Hemostasis | Dopamine receptor antagonist | Vadas et al., 1986 |
|  |  |  |  |  |  |
| (Hypoimmune, Hybrid) | Imipramine | ORM2 (.33, .21) | Neutrophil degranulation; Hemostasis | Norepinephrine reuptake inhibitor; serotonin reuptake inhibitor | Tuon et al., 2007 |
|  |  |  |  |  | Galkina et al., 2021 |
| (Hypoimmune, Hybrid) | Lidocaine | ORM2 (.33, .21) | Neutrophil degranulation; Hemostasis | Histamine receptor agonist | Schmidt et al., 1997 |
|  |  |  |  |  |  |
| (Hyperimmune, Hybrid, Hybrid, Hybrid) | Zinc acetate/Zinc chloride/Zinc sulfate | (TTR: .13, TF: .29, APP: .25, ORM2: .21) | -TTR: Neutrophil degranulation | Zinc agonist | Unoshima et al., 2001 |
|  |  |  | -Metabolism of vitamins |  | Snyder et al., 1976 |
|  |  |  | -TF: HIF-1 signaling, Ferroptosis, Post-translational protein phosphorylation |  | Newton et al., 2016 |
|  |  |  | -APP: Cytokine signaling in immune system, Post-translational protein phosphorylation, TLR cascades, Signaling by Interleukins |  |  |
|  |  |  | -ORM2: Neutrophil degranulation, hemostasis |  |  |
| Hypoimmune | Citric acid | RNASE3 (.39) | -Neutrophil degranulation | Coagulation factor antagonist | Lin et al., 2023 |
| (Hybrid, Hybrid) | Fostamatinib | (PAK1: .27, TAOK1: .01) | -PAK1: MAPK signaling, Innate Immune System, Fc gamma R-mediated phagocytosis | SYK antagonist | Liang et al., 2024 |
|  |  |  | -TAOK1: Chemokine signaling, C-type lectin receptor signaling |  |  |
| (Hypoimmune, Hybrid) | Nedocromil | FPR1 (100, 100) | -Neutrophil extracellular trap formation | Antagonist | Carolan et al., 1992 |
|  |  |  | -Neutrophil degranulation |  |  |
| (Hypoimmune, Hybrid) | (Urso)deoxycholic acid | FPR1 (100, 100) | Neutrophil extracellular trap formation; Neutrophil degranulation | Bilverdin Reductase A agonist; GPCR agonist | Miyata et al., 1999 |
|  |  |  |  |  |  |
| (Hypoimmune, Hybrid) | Rebamipide | FPR1 (100, 100) | Neutrophil extracellular trap formation; Neutrophil degranulation | Free radical scavenger | Kim et al., 2000 |
|  |  |  |  |  | Kim et al., 1999 |
| Hybrid | Curcumin | APP (.25) | Cytokine signaling in immune system; Post translational protein phosphorylation; TLR cascades; Signaling by interleukins | Cyclooxygenase inhibitor, NFKB pathway inhibitor, lipoxygenase inhibitor, histone acetyltransferase inhibitor | Kim et al., 2011 |
|  |  |  |  |  |  |
|  |  |  |  |  |  |
|  |  |  |  |  |  |
|  |  |  |  |  |  |
| Hybrid | Halothane | ATP2B1 (.01) | cGMP-PKG signaling; Calcium signaling; cAMP signaling | Antagonist | Mobert et al., 1999 |
|  |  |  |  |  |  |
|  |  |  |  |  |  |
| (Hyperimmune, Hybrid) | Spironolacctone | CACNA1G (.01, .03) | MAPK signaling | Mineralocorticoid receptor antagonist | Scbreckenberg et al., 2021 |
|  |  |  |  |  |  |
| (Hyperimmune, Hybrid) | Verapmil | CACNA1G (.01, .03) | MAPK signaling | Calcium channel blocker | Pennington et al., 1986 |
|  |  |  |  |  |  |
| (Hyperimmune, Hybrid) | Nicardipine | CACNA1G (.01, .03) | MAPK signaling | Calcium channel blocker | Shima et al., 2008 |
|  |  |  |  |  |  |
| (Hyperimmune, Hybrid) | Glutathione (disulfide) | GSTM2 (.16, .23) | Glutathione metabolism; Drug metabolism - cytochrome P450 | Agonist | Elferink et al., 1991 |
|  |  |  |  |  |  |
| (Hypoimmune, Hybrid) | Acetohydroxamic acid | MME (.20, .15) | Neutrophil degranulation; Metabolism of angiotensinogen to angiotensins | Urease inhibitor | Desouza et al., 1998 |
|  |  |  |  |  |  |
| (Hyperimmune,Hypoimmune, Hybrid) | Vitamin E | PPP2CA (.04, .14, .02) | Tight junction; Cytokine signaling in immune system; Signaling by interleukins; TLR cascades | Antioxidant | Ekstrand-Hammarstrom et al., 2007 |
|  |  |  |  |  |  |
|  |  |  |  |  |  |
|  |  |  |  |  |  |
| Hypoimmune | Pranlukast | RNASE3 (.39) | Neutrophil degranulation | Leukotriene receptor antagonist | Nishio et al., 2007 |
| Hyperimmune | Flufenamic acid | TTR (.13) | Neutrophil degranulation; Metabolism of vitamins | Chloride channel blocker | Takeuchi et al., 1998 |
|  |  |  |  |  |  |
| Hyperimmune | Dimethyl sulfoxide | TTR (.13) | Neutrophil degranulation; Metabolism of vitamins | MEK1 and MEK2 inhibitor | Choi et al., 2003 |
|  |  |  |  |  |  |
| Hybrid | Cisplatin | TF (.29) | HIF-1 signaling; Ferroptosis; Post translational protein phosphorylation | DNA synthesis inhibitor | Estrela et al., 2014 |
|  |  |  |  |  |  |
|  |  |  |  |  |  |
|  |  |  |  |  |  |
| Hybrid | Chromium | TF (.29) | HIF-1 signaling; Ferroptosis; Post translational protein phosphorylation | Binder | Sahin et al., 1996 |
|  |  |  |  |  |  |
|  |  |  |  |  |  |
|  |  |  |  |  |  |

**All FDA-approved drugs targeting differential expressed proteins regardless of neutrophil and/or sepsis affiliation**

| **Functional phenotype(s)** | **Drug(s)/small molecule(s)** | **DEP(s) and (fold change in protein expression as compared to control)** | **DEP(s) inflammatory/neutrophil pathway(s) of interest** | **Mechanism of action(s)** | **Reference(s) citing drug in sepsis and/or neutrophil study, if known** |
| --- | --- | --- | --- | --- | --- |
| (Hyperimmune, Hybrid) | Spironolactone | CACNA1G (.01, .03) | MAPK signaling; Calcium signaling | Antagonist | Al-Kadi et al.,2022 |
| (Hyperimmune, Hybrid) | Amifostine | ALPL (6.0,8.5) | Metabolic pathways; Biosynthesis of cofactors; Post-translational (protein) modification | Reducing agent | Batcik et al., 2022 |
| (Hypoimmune, Hybrid) | Chlorpromazine | ORM2 (.33, .21) | Hemostasis; Platelet degranulation; Innate immune system; Neutrophil degranulation; Response to elevated platelet cytosolic Ca2 | Inhibitor | Bertini et al., 1989 |
| (Hypoimmune, Hybrid) | Nedocromil | FPR1 (100, 100) | NETs, Rap1 signaling, Staphylococcus aureus infection | Antacid | Carol., 1992 |
| (Hypoimmune, Hybrid) | Lidocaine | ORM2 (.33, .21) | Hemostasis; Platelet degranulation; Innate immune system; Neutrophil degranulation; Response to elevated platelet cytosolic Ca2 | Agonist | Carolan, 1992 |
| Hyperimmune | Dimethyl sulfoxide | TTR (.13) | Metabolism; Extracellular matrix organization; Innate immune system; Neutrophil degranulation; Metabolism of proteins; Non-integrin mebrane-ECM interactionss | Inhibitor | Choi et al., 2003 |
| Hypoimmune | Pranlukast | RNASE3 (.39) | Asthma | Antaonist | Chu et al., 2006 |
| (Hypoimmune, Hybrid) | Cyclosporin-A | FPR1 (100, 100) | NETs, Rap1 signaling, Staphylococcus aureus infection | Inhibitor | Cockerill et al., 1995 |
| (Hyperimmune, Hybrid) | Manidipine | CACNA1G (.01, .03) | MAPK signaling; Calcium signaling | Inhibitor | Costa et al., 2010 |
| (Hyperimmune, Hybrid) | Pyridoxine | ALPL (6.0,8.5) | Metabolic pathways; Biosynthesis of cofactors; Post-translational (protein) modification | Vitamin B agonist | Deng et al., 2013 |
| (Hypoimmune, Hybrid) | Acetohydroxamic acid | MME (.20, .15) | Hematopoietic cell lineage, Innate immune system, Neutrophil degranulation | Inhibitor | Desouza et al., 1998 |
| (Hyperimmune, Hybrid) | Gabapentin | CACNA1G (.01, .03) | MAPK signaling; Calcium signaling | Agonist | Dias, 2014 |
| (Hyperimmune, Hybrid) | Gabapentin-enacarbil | CACNA1G (.01, .03) | MAPK signaling; Calcium signaling | Agonist | Dias, 2014 |
| (Hyperimmune, Hypoimmune, Hybrid) | Vitamin-E | PPP2CA (.04, .14, .02) | Cell cycle; Autophagy - other; Autophagy - animal; PI3K-Akt signaling; TGF-beta signaling; Tight junction; T cell receptor signaling; AMPK signaling? | Inhibitor | Ekstrand-Hammarstrom et al, 2007 |
| (Hyperimmune, Hybrid) | Glutathione disulfide | GSTM2 (.16, .23) | Fluid shear stress and atherosclerosis; Glutathione metabolism; Drug metabolism - cytochrome P450; Metabolic pathways; | Activator; Substrate | Elferink et al., 1991 |
| Hybrid | Cisplatin | TF (.29) | HIF-1 signaling; Ferroptosis; TGF beta signaling | DNA alkylating agent; DNA synthesis inhibitor | Estrela et al, 2014 |
| (Hypoimmune, Hybrid) | Deoxycholic acid | FPR1 (100, 100) | NETs, Rap1 signaling, Staphylococcus aureus infection | Activator | Estrela et al, 2016 |
| (Hypoimmune, Hybrid) | Sulfinpyrazone | FPR1 (100, 100) | NETs, Rap1 signaling, Staphylococcus aureus infection | Blocker/Inhibitor | Fehr et al., 1980 |
| Hyperimmune | Palmitic acid | PPT1 (5.2) | Metabolic pathways; Fatty acid elongation; Fatty acid metabolism | Activator | Feng et al., 2022 |
| Hybrid | Asparagine | ASRGL1 (6.1) | Metabolic pathways; Alanine, aspartate and glutamate metabolism; Metabolism of amino acids and derivatives; Phenylalanine metabolism | Asparagine synthase | Freund et al., 1978 |
| Hyperimmune | Diclofenac | TTR (.13) | Metabolism; Extracellular matrix organization; Innate immune system; Neutrophil degranulation; Metabolism of proteins; Non-integrin mebrane-ECM interactionss | Inhibitor | Hofbauer et al.,1999 |
| (Hypoimmune, Hybrid) | Thrombin | FGG (2.7, 8.5) | Complement and Coagulation cascade; NETs; COVID-19; Staphylococcus aureus infection | Activator | Hosac et al., 2002, Moser et al, 1990 |
| (Hypoimmune, Hybrid) | Thrombin alfa (drotrecogin alfa) | FGG (2.7, 8.5) | Complement and Coagulation cascade; NETs; COVID-19; Staphylococcus aureus infection | Activator | Hosac et al., 2002, Moser et al., 1990 |
| Hypoimmune | Methylene blue | BLVRB (.34) | Metabolic pathways; Riboflavin metabolism; Cellular response to chemical stress, Metabolism (of porphyrins); Cellular responses to stimuli | Oxidation-reduction agent | Ibarra-Estrada et al., 2023, Hong et al., 1998 |
| Hyperimmune | Thyroid, porcine | TTR (.13) | Metabolism; Extracellular matrix organization; Innate immune system; Neutrophil degranulation; Metabolism of proteins; Non-integrin mebrane-ECM interactionss | Substrate | Inan et al., 2003 |
| Hypoimmune | Flavin mononucleotide | BLVRB (.34) | Metabolic pathways; Riboflavin metabolism; Cellular response to chemical stress, Metabolism (of porphyrins); Cellular responses to stimuli | Vitamin B2 agonist | Ji et al., 2018 |
| (Hyperimmune, Hybrid) | Sargramostim | PRG2 (.05, .11) | Asthma | Agonist | Joshi et al., 2023 |
| Hyperimmune | Flufenamic acid | TTR (.13) | Metabolism; Extracellular matrix organization; Innate immune system; Neutrophil degranulation; Metabolism of proteins; Non-integrin mebrane-ECM interactionss | Activator | Kankaanranta et al., 1996 |
| Hybrid | Curcumin | APP (.25) | Cytokine signaling in immune system; Toll-like receptor 4 cascade; Toll-like receptor 3 cascade; Signaling by interleukins; Interleukin-1 family signaling; TAK1-dependent IKK and NF-kappa-B activation; Post translational protein modification; Vesicle-mediated transport; Inflammasomes; The NLRP3 inflammasome; Innate immune system; Cell recruitment (pro-inflammatory response); Diseases of programmed cell death; Metabolism of proteins | Tyrosinase inhibitor | Kim et al, 2011 |
| (Hypoimmune, Hybrid) | Rebamipide | FPR1 (100, 100) | NETs, Rap1 signaling, Staphylococcus aureus infection | Free radical scavenger | Kim et al., 2000, Kim et al., Journal of Pharmacology and Experimental Therapeutics, 1999 |
| Hypoimmune | Riboflavin | BLVRB (.34) | Metabolic pathways; Riboflavin metabolism; Cellular response to chemical stress, Metabolism (of porphyrins); Cellular responses to stimuli | Vitamin B agonist | Kim et al., Journal of Pharmacology and Experimental Therapeutics, 1999 |
| Hybrid | Gallium nitrate | TF (.29) | HIF-1 signaling; Ferroptosis; TGF beta signaling | Inhibitor | Krecic-Shepard et al., 1999 |
| (Hyperimmune, Hypoimmune, Hybrid) | Gallium citrate GA-67 | FTH1 (5.8, 2.1, 6.1) | Ferroptosis; Necroptosis; Porphyrin metabolism; Innate immune system; Neutrophil degranulation; Iron uptake and transport; Vesicle mediated transport | Binder | Kumar et al., 1975 |
| Hybrid | Gallium citrate GA-67 | TF (.29) | HIF-1 signaling; Ferroptosis; TGF beta signaling | Binder | Kumar et al., 1975 |
| Hybrid | Ferric cation | TF (.29) | HIF-1 signaling; Ferroptosis; TGF beta signaling | Agonist; binder | Kuzmicka et al., 2021 |
| Hyperimmune | Levothyroxine | TTR (.13) | Metabolism; Extracellular matrix organization; Innate immune system; Neutrophil degranulation; Metabolism of proteins; Non-integrin mebrane-ECM interactionss | Stimulant/Activator | Lado-Abeal et al., 2020 |
| Hyperimmune | Liotrix | TTR (.13) | Metabolism; Extracellular matrix organization; Innate immune system; Neutrophil degranulation; Metabolism of proteins; Non-integrin mebrane-ECM interactionss | Stimulant/Activator | Lado-Abeal et al., 2020 |
| (Hyperimmune, Hybrid) | Hyaluronic acid | HAPLN1 (7.8, 6.8) | Extracellular matrix degradation; ECM proteoglycans; | Binder | Lee et al., 2020, Forrester et al., 1982 |
| Hybrid | Fostamatinib | PAK1 (.27) | MAPK signaling; Ras signaling, cAMP signaling; Chemokine signaling; Focal adhesion; C-type lectin receptor signaling; Natural killer cell mediated cytotoxicity; T cell receptor signaling; Fc gamma R-mediated phagocytosis; Salmonella infection; Pathogenic E. coli infection | Inhibitor | Liang et al, 2024 |
| Hybrid | Fostamatinib | TAOK1 (.01) | MAPK signaling | Inhibitor | Liang et al, 2024 |
| Hypoimmune | Citric acid | RNASE3 (.39) | Asthma | Anti-chelating agent | Lin et al., 2023 |
| (Hyperimmune, Hypoimmune) | Melatonin | EPX (.28, .24) | Asthma | Inhibitor | Liu et al., 2022 |
| Hybrid | Copper | APP (.25) | Cytokine signaling in immune system; Toll-like receptor 4 cascade; Toll-like receptor 3 cascade; Signaling by interleukins; Interleukin-1 family signaling; TAK1-dependent IKK and NF-kappa-B activation; Post translational protein modification; Vesicle-mediated transport; Inflammasomes; The NLRP3 inflammasome; Innate immune system; Cell recruitment (pro-inflammatory response); Diseases of programmed cell death; Metabolism of proteins | Binder; allosteric modulator | Lominadze et al., 2004 |
| Hybrid | Copper | IDH3A (5.2) | Citric acid cycle (TCA cycle); Metabolic pathways | Binder; allosteric modulator | Lominadze et al., 2004 |
| Hyperimmune | Copper | TTR (.13) | Metabolism; Extracellular matrix organization; Innate immune system; Neutrophil degranulation; Metabolism of proteins; Non-integrin mebrane-ECM interactionss | Binder; allosteric modulator | Lominadze et al., 2004 |
| Hypoimmune | Copper | CYB5R3 (2.7) | Amino sugar and nucleotide metabolism; Innate immune system; Neutrophil degranulation; Metabolism | Binder; allosteric modulator | Lominadze et al., 2004 |
| Hybrid | Copper | TF (.29) | HIF-1 signaling; Ferroptosis; TGF beta signaling | Binder; allosteric modulator | Lominadze et al., 2004 |
| Hybrid | Tromethamine | APP (.25) | Cytokine signaling in immune system; Toll-like receptor 4 cascade; Toll-like receptor 3 cascade; Signaling by interleukins; Interleukin-1 family signaling; TAK1-dependent IKK and NF-kappa-B activation; Post translational protein modification; Vesicle-mediated transport; Inflammasomes; The NLRP3 inflammasome; Innate immune system; Cell recruitment (pro-inflammatory response); Diseases of programmed cell death; Metabolism of proteins | Protein acceptor | Lu et al., 2016 |
| Hybrid | Chromium | TF (.29) | HIF-1 signaling; Ferroptosis; TGF beta signaling | Substrate | Ma et al., 2021 |
| (Hyperimmune, Hybrid) | Cannabidiol | CACNA1G (.01, .03) | MAPK signaling; Calcium signaling | Activator? | Maayah et al., 2022, McHugh et al., 2008 |
| Hypoimmune | Cannabidiol | TRPV2 (6.0) | NOD-like receptor; Inflammatory mediator regulation of TRP channels | Activator | Maayah et al., 2022, McHugh et al., 2008 |
| Hybrid | Halothane | ATP2B1 (.01) | Calcium signaling, cGMP-PKG signaling, cAMP signaling, | Antagonist | Mobert et al., 1999 |
| (Hyperimmune, Hybrid) | Methsuximide | CACNA1G (.01, .03) | MAPK signaling; Calcium signaling | Inhibitor | NA |
| Hybrid | Manganese | IDH3A (5.2) | Citric acid cycle (TCA cycle); Metabolic pathways | Agonist | Neeli et al., 2023 |
| Hybrid | Manganese | TF (.29) | HIF-1 signaling; Ferroptosis; TGF beta signaling | Agonist | Neeli et al., 2023 |
| (Hyperimmune, Hypoimmune, Hybrid) | Iron dextran | FTH1 (5.8, 2.1, 6.1) | Ferroptosis; Necroptosis; Porphyrin metabolism; Innate immune system; Neutrophil degranulation; Iron uptake and transport; Vesicle mediated transport | Agonist | Ni et al., 2022 |
| Hybrid | Iron dextran | TF (.29) | HIF-1 signaling; Ferroptosis; TGF beta signaling | Agonist | Ni et al., 2022 |
| Hybrid | Bismuth subsalicylate | TF (.29) | HIF-1 signaling; Ferroptosis; TGF beta signaling | Antacid | Notcovich et al.,2020 |
| (Hyperimmune, Hybrid) | L-proline | PPIH (4.9, 5.3) | Infectious disease; Metabolism of RNA; SARS-CoV-1 Infection; SARS-CoV infection; Viral infection pathways | Agonist | Nunes et al., 2023 |
| (Hyperimmune, Hybrid) | Flunarizine | CACNA1G (.01, .03) | MAPK signaling; Calcium signaling | Inhibitor | Pasini et al., 1990 |
| (Hyperimmune, Hybrid) | Verapamil | CACNA1G (.01, .03) | MAPK signaling; Calcium signaling | Inhibitor | Pennington et al, 1986 |
| Hypoimmune | Flavin adenine dinucleotide | CYB5R3 (2.7) | Amino sugar and nucleotide metabolism; Innate immune system; Neutrophil degranulation; Metabolism | Agonist | Protti et al, 2006 |
| (Hypoimmune, Hybrid) | Sacubitril | MME (.20, .15) | Hematopoietic cell lineage, Innate immune system, Neutrophil degranulation | Inhibitor | Refaie et al., 2024 |
| Hybrid | Aluminum | APP (.25) | Cytokine signaling in immune system; Toll-like receptor 4 cascade; Toll-like receptor 3 cascade; Signaling by interleukins; Interleukin-1 family signaling; TAK1-dependent IKK and NF-kappa-B activation; Post translational protein modification; Vesicle-mediated transport; Inflammasomes; The NLRP3 inflammasome; Innate immune system; Cell recruitment (pro-inflammatory response); Diseases of programmed cell death; Metabolism of proteins | Astringent | Reithhofer et al., 2020 |
| Hybrid | Aluminum | TF (.29) | HIF-1 signaling; Ferroptosis; TGF beta signaling | Astringent | Reithhofer et al., 2021 |
| (Hyperimmune, Hybrid) | Benidipine | CACNA1G (.01, .03) | MAPK signaling; Calcium signaling | Inhibitor | Shima et al., 2008 |
| (Hyperimmune, Hybrid) | Nicardipine | CACNA1G (.01, .03) | MAPK signaling; Calcium signaling | Inhibitor | Shima et al., 2008 |
| Hypoimmune | Amiloride | TRPV2 (6.0) | NOD-like receptor; Inflammatory mediator regulation of TRP channels | Inhibitor | Sikes et al., 2005 |
| (Hyperimmune, Hybrid) | Strontium chloride | ALPL (6.0,8.5) | Metabolic pathways; Biosynthesis of cofactors; Post-translational (protein) modification | Inhibitor | Soylu et al., 2018 |
| Hybrid | L-aspartic acid | ASRGL1 (6.1) | Metabolic pathways; Alanine, aspartate and glutamate metabolism; Metabolism of amino acids and derivatives; Phenylalanine metabolism | Substrate | Su et al., 2015 |
| (Hyperimmune, Hybrid) | Glutathione | GSTM2 (.16, .23) | Fluid shear stress and atherosclerosis; Glutathione metabolism; Drug metabolism - cytochrome P450; Metabolic pathways; | Antioxidant | Tandon et al., 2024 |
| Hypoimmune | Riboflavin 5 phosphate sodium | BLVRB (.34) | Metabolic pathways; Riboflavin metabolism; Cellular response to chemical stress, Metabolism (of porphyrins); Cellular responses to stimuli | Activator | Toyosawa et al., 2004 |
| (Hypoimmune, Hybrid) | Imipramine | ORM2 (.33, .21) | Hemostasis; Platelet degranulation; Innate immune system; Neutrophil degranulation; Response to elevated platelet cytosolic Ca2 | Inhibitor | Tuon et al., 2007, Galkina et al., 2021 |
| Hybrid | Aducanumab | APP (.25) | Cytokine signaling in immune system; Toll-like receptor 4 cascade; Toll-like receptor 3 cascade; Signaling by interleukins; Interleukin-1 family signaling; TAK1-dependent IKK and NF-kappa-B activation; Post translational protein modification; Vesicle-mediated transport; Inflammasomes; The NLRP3 inflammasome; Innate immune system; Cell recruitment (pro-inflammatory response); Diseases of programmed cell death; Metabolism of proteins | Antagonist; Binder | Unknown |
| Hybrid | Aluminum acetate | APP (.25) | Cytokine signaling in immune system; Toll-like receptor 4 cascade; Toll-like receptor 3 cascade; Signaling by interleukins; Interleukin-1 family signaling; TAK1-dependent IKK and NF-kappa-B activation; Post translational protein modification; Vesicle-mediated transport; Inflammasomes; The NLRP3 inflammasome; Innate immune system; Cell recruitment (pro-inflammatory response); Diseases of programmed cell death; Metabolism of proteins | Astringent | Unknown |
| Hybrid | Aluminum acetate | TF (.29) | HIF-1 signaling; Ferroptosis; TGF beta signaling | Astringent | Unknown |
| Hybrid | Aluminum phosphate | APP (.25) | Cytokine signaling in immune system; Toll-like receptor 4 cascade; Toll-like receptor 3 cascade; Signaling by interleukins; Interleukin-1 family signaling; TAK1-dependent IKK and NF-kappa-B activation; Post translational protein modification; Vesicle-mediated transport; Inflammasomes; The NLRP3 inflammasome; Innate immune system; Cell recruitment (pro-inflammatory response); Diseases of programmed cell death; Metabolism of proteins | Astringent | Unknown |
| Hybrid | Aluminum phosphate | TF (.29) | HIF-1 signaling; Ferroptosis; TGF beta signaling | Astringent | Unknown |
| Hybrid | Chromic nitrate | TF (.29) | HIF-1 signaling; Ferroptosis; TGF beta signaling | Substrate | Unknown |
| Hybrid | Chromium gluconate | TF (.29) | HIF-1 signaling; Ferroptosis; TGF beta signaling | Substrate | Unknown |
| Hybrid | Chromium nicotinate | TF (.29) | HIF-1 signaling; Ferroptosis; TGF beta signaling | Substrate | Unknown |
| Hybrid | Chromous sulfate | TF (.29) | HIF-1 signaling; Ferroptosis; TGF beta signaling | Substrate | Unknown |
| Hyperimmune | Diflunisal | TTR (.13) | Metabolism; Extracellular matrix organization; Innate immune system; Neutrophil degranulation; Metabolism of proteins; Non-integrin mebrane-ECM interactionss | Inhibitor | Unknown |
| Hybrid | Dimercaprol | APP (.25) | Cytokine signaling in immune system; Toll-like receptor 4 cascade; Toll-like receptor 3 cascade; Signaling by interleukins; Interleukin-1 family signaling; TAK1-dependent IKK and NF-kappa-B activation; Post translational protein modification; Vesicle-mediated transport; Inflammasomes; The NLRP3 inflammasome; Innate immune system; Cell recruitment (pro-inflammatory response); Diseases of programmed cell death; Metabolism of proteins | Chelating agent | Unknown |
| (Hypoimmune, Hybrid) | Disopyramide | ORM2 (.33, .21) | Hemostasis; Platelet degranulation; Innate immune system; Neutrophil degranulation; Response to elevated platelet cytosolic Ca2 | Inhibitor | Unknown |
| (Hyperimmune, Hybrid) | Eliglustat | UGCG (.20, .24) | Metabolic pathways; Sphingolipid metabolism | Inhibitor | Unknown |
| (Hyperimmune, Hybrid) | Ethosuximide | CACNA1G (.01, .03) | MAPK signaling; Calcium signaling | Succinimide antiepileptic | Unknown |
| (Hyperimmune, Hypoimmune, Hybrid) | Ferric pyrophosphate citrate | FTH1 (5.8, 2.1, 6.1) | Ferroptosis; Necroptosis; Porphyrin metabolism; Innate immune system; Neutrophil degranulation; Iron uptake and transport; Vesicle mediated transport | Agonist; binder | Unknown |
| (Hyperimmune, Hypoimmune, Hybrid) | Ferrous ascorbate | FTH1 (5.8, 2.1, 6.1) | Ferroptosis; Necroptosis; Porphyrin metabolism; Innate immune system; Neutrophil degranulation; Iron uptake and transport; Vesicle mediated transport | Agonist; binder | Unknown |
| Hybrid | Ferrous ascorbate | TF (.29) | HIF-1 signaling; Ferroptosis; TGF beta signaling | Agonist; binder | Unknown |
| (Hyperimmune, Hypoimmune, Hybrid) | Ferrous fumarate | FTH1 (5.8, 2.1, 6.1) | Ferroptosis; Necroptosis; Porphyrin metabolism; Innate immune system; Neutrophil degranulation; Iron uptake and transport; Vesicle mediated transport | Agonist; binder | Unknown |
| Hybrid | Ferrous fumarate | TF (.29) | HIF-1 signaling; Ferroptosis; TGF beta signaling | Agonist; binder | Unknown |
| (Hyperimmune, Hypoimmune, Hybrid) | Ferrous gluconate | FTH1 (5.8, 2.1, 6.1) | Ferroptosis; Necroptosis; Porphyrin metabolism; Innate immune system; Neutrophil degranulation; Iron uptake and transport; Vesicle mediated transport | Agonist; binder | Unknown |
| Hybrid | Ferrous gluconate | TF (.29) | HIF-1 signaling; Ferroptosis; TGF beta signaling | Agonist; binder | Unknown |
| (Hyperimmune, Hypoimmune, Hybrid) | Ferrous glycine sulfate | FTH1 (5.8, 2.1, 6.1) | Ferroptosis; Necroptosis; Porphyrin metabolism; Innate immune system; Neutrophil degranulation; Iron uptake and transport; Vesicle mediated transport | Agonist; binder | Unknown |
| Hybrid | Ferrous glycine sulfate | TF (.29) | HIF-1 signaling; Ferroptosis; TGF beta signaling | Agonist; binder | Unknown |
| (Hyperimmune, Hypoimmune, Hybrid) | Ferrous succinate | FTH1 (5.8, 2.1, 6.1) | Ferroptosis; Necroptosis; Porphyrin metabolism; Innate immune system; Neutrophil degranulation; Iron uptake and transport; Vesicle mediated transport | Agonist; binder | Unknown |
| Hybrid | Ferrous succinate | TF (.29) | HIF-1 signaling; Ferroptosis; TGF beta signaling | Agonist; binder | Unknown |
| Hybrid | Florbetaben F18 | APP (.25) | Cytokine signaling in immune system; Toll-like receptor 4 cascade; Toll-like receptor 3 cascade; Signaling by interleukins; Interleukin-1 family signaling; TAK1-dependent IKK and NF-kappa-B activation; Post translational protein modification; Vesicle-mediated transport; Inflammasomes; The NLRP3 inflammasome; Innate immune system; Cell recruitment (pro-inflammatory response); Diseases of programmed cell death; Metabolism of proteins | Binder | Unknown |
| Hybrid | Florbetapir | APP (.25) | Cytokine signaling in immune system; Toll-like receptor 4 cascade; Toll-like receptor 3 cascade; Signaling by interleukins; Interleukin-1 family signaling; TAK1-dependent IKK and NF-kappa-B activation; Post translational protein modification; Vesicle-mediated transport; Inflammasomes; The NLRP3 inflammasome; Innate immune system; Cell recruitment (pro-inflammatory response); Diseases of programmed cell death; Metabolism of proteins | Binder | Unknown |
| Hybrid | Lecanemab | APP (.25) | Cytokine signaling in immune system; Toll-like receptor 4 cascade; Toll-like receptor 3 cascade; Signaling by interleukins; Interleukin-1 family signaling; TAK1-dependent IKK and NF-kappa-B activation; Post translational protein modification; Vesicle-mediated transport; Inflammasomes; The NLRP3 inflammasome; Innate immune system; Cell recruitment (pro-inflammatory response); Diseases of programmed cell death; Metabolism of proteins | Binder; Amyloid beta targeting antibody | Unknown |
| Hyperimmune | Liothyronine | TTR (.13) | Metabolism; Extracellular matrix organization; Innate immune system; Neutrophil degranulation; Metabolism of proteins; Non-integrin mebrane-ECM interactionss | Stimulant/Activator | Unknown |
| (Hyperimmune, Hybrid) | Miglustat | UGCG (.20, .24) | Metabolic pathways; Sphingolipid metabolism | Inhibitor | Unknown |
| (Hyperimmune, Hybrid) | Orlistat | DAGLB (10.0, 12.1) | Hemostasis; Platelet activation, signaling and aggregation | Inhibitor | Unknown |
| (Hyperimmune, Hybrid) | Paramethadione | CACNA1G (.01, .03) | MAPK signaling; Calcium signaling | Inhibitor | Unknown |
| (Hypoimmune, Hybrid) | Racecadotril | MME (.20, .15) | Hematopoietic cell lineage, Innate immune system, Neutrophil degranulation | Inhibitor | Unknown |
| Hybrid | Rose bengal | TF (.29) | HIF-1 signaling; Ferroptosis; TGF beta signaling | Agonist | Unknown |
| Hyperimmune | Tafamidis | TTR (.13) | Metabolism; Extracellular matrix organization; Innate immune system; Neutrophil degranulation; Metabolism of proteins; Non-integrin mebrane-ECM interactionss | Inhibitor | Unknown |
| Hyperimmune | Tafamidis-meglumine | TTR (.13) | Metabolism; Extracellular matrix organization; Innate immune system; Neutrophil degranulation; Metabolism of proteins; Non-integrin mebrane-ECM interactionss | Inhibitor | Unknown |
| Hybrid | tatraferric tricitrate decahydrate | TF (.29) | HIF-1 signaling; Ferroptosis; TGF beta signaling | Ligand | Unknown |
| (Hyperimmune, Hybrid) | Trimethadione | CACNA1G (.01, .03) | MAPK signaling; Calcium signaling | Inhibitor | Unknown |
| (Hyperimmune, Hypoimmune) | Zopiclone | TSPO (100, 100) | Cholesterol metabolism; Human T cell leukemia virus 1 infection | Activator | Unknown |
| (Hypoimmune, Hybrid) | Zinc | ORM2 (.33, .21) | Hemostasis; Platelet degranulation; Innate immune system; Neutrophil degranulation; Response to elevated platelet cytosolic Ca2 | Cofactor | Unoshima et al.,2001, Snyder et al., 1976, Newton et al., 2016 |
| Hybrid | Zinc | TF (.29) | HIF-1 signaling; Ferroptosis; TGF beta signaling | Cofactor | Unoshima et al.,2001, Snyder et al., 1976, Newton et al., 2016 |
| Hyperimmune | Zinc | TTR (.13) | Metabolism; Extracellular matrix organization; Innate immune system; Neutrophil degranulation; Metabolism of proteins; Non-integrin mebrane-ECM interactionss | Cofactor | Unoshima et al.,2001, Snyder et al., 1976, Newton et al., 2016 |
| (Hypoimmune, Hybrid) | Zinc acetate | ORM2 (.33, .21) | Hemostasis; Platelet degranulation; Innate immune system; Neutrophil degranulation; Response to elevated platelet cytosolic Ca2 | Agonist | Unoshima et al.,2001, Snyder et al., 1976, Newton et al., 2016 |
| Hybrid | Zinc acetate | APP (.25) | Cytokine signaling in immune system; Toll-like receptor 4 cascade; Toll-like receptor 3 cascade; Signaling by interleukins; Interleukin-1 family signaling; TAK1-dependent IKK and NF-kappa-B activation; Post translational protein modification; Vesicle-mediated transport; Inflammasomes; The NLRP3 inflammasome; Innate immune system; Cell recruitment (pro-inflammatory response); Diseases of programmed cell death; Metabolism of proteins | Agonist | Unoshima et al.,2001, Snyder et al., 1976, Newton et al., 2016 |
| Hybrid | Zinc acetate | TF (.29) | HIF-1 signaling; Ferroptosis; TGF beta signaling | Agonist | Unoshima et al.,2001, Snyder et al., 1976, Newton et al., 2016 |
| Hyperimmune | Zinc acetate | TTR (.13) | Metabolism; Extracellular matrix organization; Innate immune system; Neutrophil degranulation; Metabolism of proteins; Non-integrin mebrane-ECM interactionss | Agonist | Unoshima et al.,2001, Snyder et al., 1976, Newton et al., 2016 |
| (Hypoimmune, Hybrid) | Zinc chloride | ORM2 (.33, .21) | Hemostasis; Platelet degranulation; Innate immune system; Neutrophil degranulation; Response to elevated platelet cytosolic Ca2 | Ligand | Unoshima et al.,2001, Snyder et al., 1976, Newton et al., 2016 |
| Hybrid | Zinc chloride | APP (.25) | Cytokine signaling in immune system; Toll-like receptor 4 cascade; Toll-like receptor 3 cascade; Signaling by interleukins; Interleukin-1 family signaling; TAK1-dependent IKK and NF-kappa-B activation; Post translational protein modification; Vesicle-mediated transport; Inflammasomes; The NLRP3 inflammasome; Innate immune system; Cell recruitment (pro-inflammatory response); Diseases of programmed cell death; Metabolism of proteins | Ligand | Unoshima et al.,2001, Snyder et al., 1976, Newton et al., 2016 |
| Hybrid | Zinc chloride | TF (.29) | HIF-1 signaling; Ferroptosis; TGF beta signaling | Ligand | Unoshima et al.,2001, Snyder et al., 1976, Newton et al., 2016 |
| Hyperimmune | Zinc chloride | TTR (.13) | Metabolism; Extracellular matrix organization; Innate immune system; Neutrophil degranulation; Metabolism of proteins; Non-integrin mebrane-ECM interactionss | Ligand | Unoshima et al.,2001, Snyder et al., 1976, Newton et al., 2016 |
| (Hypoimmune, Hybrid) | Zinc sulfate | ORM2 (.33, .21) | Hemostasis; Platelet degranulation; Innate immune system; Neutrophil degranulation; Response to elevated platelet cytosolic Ca2 | Inhibitor | Unoshima et al.,2001, Snyder et al., 1976, Newton et al., 2016 |
| Hybrid | Zinc sulfate | APP (.25) | Cytokine signaling in immune system; Toll-like receptor 4 cascade; Toll-like receptor 3 cascade; Signaling by interleukins; Interleukin-1 family signaling; TAK1-dependent IKK and NF-kappa-B activation; Post translational protein modification; Vesicle-mediated transport; Inflammasomes; The NLRP3 inflammasome; Innate immune system; Cell recruitment (pro-inflammatory response); Diseases of programmed cell death; Metabolism of proteins | Inhibitor | Unoshima et al.,2001, Snyder et al., 1976, Newton et al., 2016 |
| Hybrid | Zinc sulfate | TF (.29) | HIF-1 signaling; Ferroptosis; TGF beta signaling | Inhibitor | Unoshima et al.,2001, Snyder et al., 1976, Newton et al., 2016 |
| Hyperimmune | Zinc sulfate | TTR (.13) | Metabolism; Extracellular matrix organization; Innate immune system; Neutrophil degranulation; Metabolism of proteins; Non-integrin mebrane-ECM interactionss | Inhibitor | Unoshima et al.,2001, Snyder et al., 1976, Newton et al., 2016 |
| (Hyperimmune, Hybrid) | Zonisamide | CACNA1G (.01, .03) | MAPK signaling; Calcium signaling | Inhibitor | Unoshima et al.,2001, Snyder et al., 1976, Newton et al., 2016 |
| Hybrid | Deferozamine | APP (.25) | Cytokine signaling in immune system; Toll-like receptor 4 cascade; Toll-like receptor 3 cascade; Signaling by interleukins; Interleukin-1 family signaling; TAK1-dependent IKK and NF-kappa-B activation; Post translational protein modification; Vesicle-mediated transport; Inflammasomes; The NLRP3 inflammasome; Innate immune system; Cell recruitment (pro-inflammatory response); Diseases of programmed cell death; Metabolism of proteins | Chelating agent | Vlahakos et al., 2012 |
| Hybrid | Ferrous sulfate anhydrous | TF (.29) | HIF-1 signaling; Ferroptosis; TGF beta signaling | Substrate | Walter et al., 1986 |
| (Hypoimmune, Hybrid) | Alteplase | FGG (2.7, 8.5) | Complement and Coagulation cascade; NETs; COVID-19; Staphylococcus aureus infection | Binder | Wang et al., 2021 |
| (Hyperimmune, Hypoimmune, Hybrid) | Iron | FTH1 (5.8, 2.1, 6.1) | Ferroptosis; Necroptosis; Porphyrin metabolism; Innate immune system; Neutrophil degranulation; Iron uptake and transport; Vesicle mediated transport | Cofactor | Xia et al., 2016 |
| Hybrid | Iron | TF (.29) | HIF-1 signaling; Ferroptosis; TGF beta signaling | Cofactor | Xia et al., 2016 |
| Hybrid | Ferumoxytol | TF (.29) | HIF-1 signaling; Ferroptosis; TGF beta signaling | Agonist | Xue et al., 2019 |
| (Hyperimmune, Hybrid) | NADH | PDHA1 (6.5, 8.1) | Glycolysis/Gluconeogensis; Citric cycle (TCA cycle); Pyruvate metabolism; Metabolic pathways; HIF-1 signaling; Lipoic acid metabolism | Agonist | Ye et al., 2022 |
| Hybrid | NADH | IDH3A (5.2) | Citric acid cycle (TCA cycle); Metabolic pathways | Agonist | Ye et al., 2022 |
| Hypoimmune | NADH | BLVRB (.34) | Metabolic pathways; Riboflavin metabolism; Cellular response to chemical stress, Metabolism (of porphyrins); Cellular responses to stimuli | Agonist | Ye et al., 2022 |
| Hypoimmune | NADH | DHCR7 (.28) | Metabolic pathways; Metabolism of steroids; Metabolism of lipids | Agonist | Ye et al., 2022 |
| Hypoimmune | NADH | HIBADH (.01) | Metabolic pathways | Agonist | Ye et al., 2022 |
| Hypoimmune | NADH | NDUFA2 (.21) | Oxidative phosphorylation; Metabolic pathways; Aerobic respitation and respiratory electron transport; Metabolism of proteins; Respiratory electron transport; Mitochondrial protein degradation | Agonist | Ye et al., 2022 |
| Hypoimmune | NADH | NDUFV2 (5.1) | Oxidative phosphorylation; Metabolic pathways | Agonist | Ye et al., 2022 |
| Hypoimmune | NADH | CYB5R3 (2.7) | Amino sugar and nucleotide metabolism; Innate immune system; Neutrophil degranulation; Metabolism | Agonist | Ye et al., 2022 |

**(Pre)clinical trial therapeutics targeting the differential expressed proteins, regardless of sepsis and/or neutrophil affiliation**

| **Functional phenotype(s)** | **Drug(s)/small molecule(s), if name is specifically known** | **DEP(s) and (fold change in protein expression as compared to control)** | **DEP(s) inflammatory/neutrophil pathway(s) of interest** | **Mechanism of action(s), if applicable** | **Reference citing drug in sepsis and/or neutrophil study, if know** |
| --- | --- | --- | --- | --- | --- |
| Hybrid | Unknown | VTN (11) | PI3K-Akt signaling | Inhibitor | Bae et al., 2012 |
| (Hyperimmune, Hybrid) | ISO-1 | MIF (6, 5) | Tyrosine metabolism, Phenylalanine metabolism, Metabolic pathways | Macrophage migration inhibiting factor inhibitor | Maayah et al., 2022 Yang et al., 2022 |
| (Hyperimmune, Hybrid) | YZ9 | MIF (6, 5) | Tyrosine metabolism, Phenylalanine metabolism, Metabolic pathways | Phosphofructokinase inhibitor | McHugh et al., 2008 |
| (Hyperimmune, Hybrid) | 4-iodo-6-phenylpyrimidine | MIF (6, 5) | Tyrosine metabolism, Phenylalanine metabolism, Metabolic pathways, SCI | Macrophage migration inhibiting factor inhibitor | Protti et al., 2006 |
| (Hyperimmune, Hybrid) | Unknown | ALG1 (35, 12) | Post translation protein modification | Unknown | Unknown |
| (Hyperimmune, Hybrid) | Unknown | EXOC8 (.085, .075) | Vesicle mediated transport | Unknown | Unknown |
| (Hyperimmune, Hybrid) | Unknown | GNB1 (.038, .04) | Chemokine signaling, Ras signaling, PI3K-Akt signaling, ADORA2B mediated anti inflammatory cytokines production, G beta gamma signaling, Inhibition of voltae gated Ca2+ channels via Gbeta/gamma subunits | Unknown | Unknown |
| (Hyperimmune, Hybrid) | Chicago-sky-blue-6b | MIF (6, 5) | Tyrosine metabolism, Phenylalanine metabolism, Metabolic pathways | Glutamate inhibitor; macrophage inhibiting factor inhibitor | Unknown |
| (Hyperimmune, Hybrid) | Citric acid | MIF (6, 5) | Tyrosine metabolism, Phenylalanine metabolism, Metabolic pathways | Coagulation factor inhibitor | Unknown |
| (Hyperimmune, Hybrid) | CPSI-1306-(+/-) | MIF (6, 5) | Tyrosine metabolism, Phenylalanine metabolism, Metabolic pathways | Macrophage migration inhibiting factor inhibitor | Unknown |
| (Hyperimmune, Hybrid) | 7-Hydroxy-2-Oxo-Chromene-3-Carboxylic Acid Ethyl Ester | MIF (6, 5) | Tyrosine metabolism, Phenylalanine metabolism, Metabolic pathways | Macrophage migration inhibiting factor inhibitor | Unknown |
| (Hyperimmune, Hybrid) | 3,4-Dihydroxycinnamic Acid | MIF (6, 5) | Tyrosine metabolism, Phenylalanine metabolism, Metabolic pathways | Macrophage migration inhibiting factor inhibitor; Histidine ammonia-lyase | Unknown |
| (Hyperimmune, Hybrid) | 4-Hydroxyphenylpyruvic acid | MIF (6, 5) | Tyrosine metabolism, Phenylalanine metabolism, Metabolic pathways | Macrophage migration inhibiting factor inhibitor; Prephenate dehydrogenase | Unknown |
| (Hyperimmune, Hybrid) | 3-(4-hydroxyphenyl)-4,5-dihydro-5-isoxazole-acetic acid methyl ester | MIF (6, 5) | Tyrosine metabolism, Phenylalanine metabolism, Metabolic pathways | Macrophage migration inhibiting factor inhibitor | Unknown |
| (Hyperimmune, Hybrid) | 4-hydroxybenzaldehyde O-(cyclohexylcarbonyl)oxime | MIF (6, 5) | Tyrosine metabolism, Phenylalanine metabolism, Metabolic pathways | Macrophage migration inhibiting factor inhibitor | Unknown |
| (Hyperimmune, Hybrid) | 3-fluoro-4-hydroxybenzaldehyde O-(cyclohexylcarbony)oxime | MIF (6, 5) | Tyrosine metabolism, Phenylalanine metabolism, Metabolic pathways | Macrophage migration inhibiting factor inhibitor | Unknown |
| (Hyperimmune, Hybrid) | 4-hydroxybenzaldehyde O-(3,3-dimethylbutanoyl)oxime | MIF (6, 5) | Tyrosine metabolism, Phenylalanine metabolism, Metabolic pathways | Macrophage migration inhibiting factor inhibitor | Unknown |
| (Hyperimmune, Hybrid) | 6-hydroxy-1,3-benzothiazole-2-sulfonamide | MIF (6, 5) | Tyrosine metabolism, Phenylalanine metabolism, Metabolic pathways | Macrophage migration inhibiting factor inhibitor; Carbonic anhydrase 2 | Unknown |
| (Hyperimmune, Hybrid) | CU-T12-9 | TLR1 (100, 100) | Unknown | Toll like receptor agonist | Unknown |
| (Hyperimmune, Hybrid) | Unknown | TPP1 (9, 7) | Lysosome, Cell response to stimuli (stress), Unfolded protein response, VEGFA-VEGFR2 signaling | Unknown | Unknown |
| (Hyperimmune, Hypoimmune, Hybrid) | EED226 | EED (100, 100, 100) | Polycomb repressive complex, Viral infection pathways, Defective pyroptosis, Diseases of programmed cell death, Infectious disease, Intracellular signaling by second messengers | Polycomb protein inhibitor | Unknown |
| (Hypoimmune, Hybrid) | Unknown | DDX3Y (.01, .095) | Unknown | Unknown | Unknown |
| Hybrid | Unknown | FLNC (.32) | Unknown | Unknown | Unknown |
| Hybrid | Unknown | UBE4A (.095) | Ubiiquitin mediated proteolysis, Adaptive immune system, Antigen processing: uniquitination & proteasome degradation, Class I MHC mediated antigen processing and presentation | Unknown | Unknown |
| Hyperimmune | Casin | CDC42 (.17) | MAPL signaling, Ras signaling, Chemoskine signaling, VEGF signaling, Adherens junction, T cell receptor signaling, Leukocyte transendothelial migration, Regulation of actin cytoskeleton, AGE-RAGE signaling, Bacterial invasion of epithelial cells, Lipid and atherosclerosis, Hemostasis, Cytokine signaling in immune system, Innate immune system, Signaling by VEGF, Signaling by EGFR, VEGFA-VEGFR2 pathway, IL-12 family signaling, MAPK family signaling, Gene and protein expression by JAK-STAT signaling after IL-12 stimulation, G beta gamma signaling through CDC42 | GTPase inhibitor | Unknown |
| Hyperimmune | ML141 | CDC42 (.17) | MAPL signaling, Ras signaling, Chemoskine signaling, VEGF signaling, Adherens junction, T cell receptor signaling, Leukocyte transendothelial migration, Regulation of actin cytoskeleton, AGE-RAGE signaling, Bacterial invasion of epithelial cells, Lipid and atherosclerosis | GTPase inhibitor | Unknown |
| Hyperimmune | RKI-1447 | CDC42 (.17) | MAPL signaling, Ras signaling, Chemoskine signaling, VEGF signaling, Adherens junction, T cell receptor signaling, Leukocyte transendothelial migration, Regulation of actin cytoskeleton, AGE-RAGE signaling, Bacterial invasion of epithelial cells, Lipid and atherosclerosis | Rho associated kinase inhibitor | Unknown |
| Hyperimmune | ZCL-278 | CDC42 (.17) | MAPL signaling, Ras signaling, Chemoskine signaling, VEGF signaling, Adherens junction, T cell receptor signaling, Leukocyte transendothelial migration, Regulation of actin cytoskeleton, AGE-RAGE signaling, Bacterial invasion of epithelial cells, Lipid and atherosclerosis | CDC inhibitor | Unknown |
| Hyperimmune | Aminophosphonic acid-guanylate ester | CDC42 (.17) | MAPL signaling, Ras signaling, Chemoskine signaling, VEGF signaling, Adherens junction, T cell receptor signaling, Leukocyte transendothelial migration, Regulation of actin cytoskeleton, AGE-RAGE signaling, Bacterial invasion of epithelial cells, Lipid and atherosclerosis | Ubiquitin protein ligase activity | Unknown |
| Hyperimmune | Guanosine-5' Diphosphate | CDC42 (.17) | MAPL signaling, Ras signaling, Chemoskine signaling, VEGF signaling, Adherens junction, T cell receptor signaling, Leukocyte transendothelial migration, Regulation of actin cytoskeleton, AGE-RAGE signaling, Bacterial invasion of epithelial cells, Lipid and atherosclerosis | Unknown | Unknown |
| Hyperimmune | Unknown | LRSAM1 (7.0) | Adaptive immune system, Antigen processing, Ubiquitination and proteolysis | Unknown | Unknown |
| Hyperimmune | Unknown | MBOAT7 (.01) | Glycerophospholipid metabolism, Mtabolism of lipids, Phospholipid metabolism | Unknown | Unknown |
| Hyperimmune | Elafin | PRTN3 (4.9) | Hemostasis, Cytokine signaling in immune system, Innate immune system, Neutrophil degranulation, Antimicrobial peptides, Signaling by interleukins | Inhibitor | Unknown |
| Hyperimmune | Unknown | SP110 (.01) | Unknown | Unknown | Unknown |
| Hyperimmune | Unknown | SPPL2A (.07) | Regulation of TNFR1 signaling, Death receptor signaling, TNF signaling | Unknown | Unknown |
| Hyperimmune, Hypoimmune, Hybrid | Unknown | CD74 (45, 8, 8) | Antigen processing and presentation; Adaptive immune system, Cell surface interactions at the vascular wall, MHC class II antigen presentation | Unknown | Unknown |
| Hypoimmune | Unknown | BAZ1B (.32) | ATP dependent chromatin remodeling, Epigenetic regulation of gene expression, | Unknown | Unknown |
| Hypoimmune | Unknown | MIA3 (.19) | Post translation protein modification, Vesicle-mediated transport, | Unknown | Unknown |
| Hypoimmune | Unknown | PDP1 (.01) | TCA cycle and respiratory electron transport, Pyruvate metabolism, | Unknown | Unknown |
| Hypoimmune | Unknown | PMPCA (.01) | Mitochondrial protein import; Mitochondrial calcium ion transport, Mitochondrial protein degradation | Unknown | Unknown |
| Hypoimmune | BQU57 | RALA (4.2) | Ras signaling, Rap1 signaling, phospholipase D signaling, cytokine signaling in immune ystem, IL12 signaling, JAK-STAT signaling after IL-12 stimulation | RAS GTPase inhibitor | Unknown |
| Hypoimmune | RBC8 | RALA (4.2) | Ras signaling, Rap1 signaling, phospholipase D signaling | Ral GTPase inhibitor | Unknown |
| Hypoimmune | Guanosine-5' Diphosphate | RALA (4.2) | Ras signaling, Rap1 signaling, phospholipase D signaling | Unknown | Unknown |
| Hyperimmune | Ploysialic acid | H2AC21 (2) | Neutrophil extracellular trap formation | Inhibitor | Y. Li et al., 2021 |
| Hyperimmune | Heparin | H2AC21 (2) | Neutrophil extracellular trap formation | Inhibitor | Y. Li et al., 2021 |
| Hyperimmune | C-reactive protein | H2AC21 (2) | Neutrophil extracellular trap formation | Inhibitor | Y. Li et al., 2021 |
| Hyperimmune | Tirofiban | H2AC21 (2) | Neutrophil extracellular trap formation | Inhibitor | Y. Li et al., 2021 |
| Hyperimmune | Thrombomodulin/activated protein C | H2AC21 (2) | Neutrophil extracellular trap formation | Inhibitor | Y. Li et al., 2021 |
| (Hyperimmune, Hybrid) | Caffeic-acid | MIF (6, 5) | Tyrosine metabolism, Phenylalanine metabolism, Metabolic pathways, Cytokine sigaling in immune system, Innate immune system, Neutrophil degranulation, Cell surface interactions at vascular wall, IL-12 signaling, | HIV integrase inhibitor; lipoxygenase inhibitor; nitric oxide production inhibitor tumor necrosis factor production inhibitor | Ye et al., 2022 |

**References**

Ainosah, R., Hagras, M., Alharthi, S., & Saadah, O. (2020). The effects of ursodeoxycholic acid on sepsis-induced cholestasis management in an animal model. *Journal of Taibah University Medical Sciences*, *15*, 312-320. <https://doi.org/10.1016/j.jtumed.2020.04.007>

Al-Kadi, A., El-Daly, M., El-Tahawy, N. F. G., Khalifa, M. M. A., & Ahmed, A. S. F. (2022). Angiotensin aldosterone inhibitors improve survival and ameliorate kidney injury induced by sepsis through suppression of inflammation and apoptosis. *Fundamental & Clinical Pharmacology*, *36*(2), 286-295. <https://doi.org/10.1111/fcp.12718>

Bae, H., Zmijewski, J., Deshane, J., Zhi, D., Thompson, L., Peterson, C.,…Abraham, E. (2012). Vitronectin Inhibits Neutrophil Apoptosis through Activation of Integrin-Associated Signaling Pathways. *American Journal of Respiratory Cellular and Molecular Biology*, *46*, 790-796. <https://doi.org/10.1165/rcmb.2011-0187OC>

Batcik, S., Tumkaya, L., Mercantepe, T., Atak, M., Topcu, A., Uydu, H. A., & Mercantepe, F. (2022). The nephroprotective effect of amifostine in a cecal ligation-induced sepsis model in terms of oxidative stress and inflammation. *European Review for Medical and Pharmacological Sciences*, *26*(24), 9144-9156.

Carolan, E. J., & Casale, T. B. (1992). Effects of Nedocromil Sodium and WEB-2086 on Chemoattractant-Stimulated Neutrophil Migration Through Cellular and Noncellular Barriers. *Annals of Allergy*, *69*(4), 323-328.

Choi, K. S., Garyu, J., Park, J., & Dumler, J. S. (2003). Diminished adhesion of Anaplasma phagocytophilum-infected neutrophils to endothelial cells is associated with reduced expression of leukocyte surface selectin. *Infection and Immunity*, *71*(8), 4586-4594. <https://doi.org/10.1128/iai.71.8.4586-4594.2003>

Chu, L., Wei, E., Yu, G., Fang, S., Zhou, Y., Wang, M., & Zhang, W. (2006). Pranlukast reduces neutrophil but not macrophage/microglial accumulation in brain after focal cerebral ischemia in mice. *Acta Pharmacologica Sinica*, *27*, 282-288. <https://doi.org/10.1111/j.1745-7254.2006.00290.x>

Cockerill, G., Bert, A., Ryan, G., Gamble, J., Vadas, M., & Cockerill, P. (1995). Regulation of Granulocyte-Macrophage Colony-Stimulating Factor and E-Selectin Expression in Endothelial-Cells by Cyclosporine-A and the T-cell Transcription Factor NFat. *Blood*, *86*, 2689-2698.

Costa, S., Zimetti, F., Pedrelli, M., Cremonesi, G., & Bernini, F. (2010). Manidipine reduces pro-inflammatory cytokines secretion in human endothelial cells and macrophages. *Pharmacological Research*, *62*, 265-270. <https://doi.org/10.1016/j.phrs.2010.03.004>

Dahinden, C., & Fehr, J. (1980). Receptor-Directed Inhibition of Chemotactic Factor-Induced Neutrophil Hyperactivity by Pyrazolon Derivatives - Definition of a Chemotactic Peptide Antagonist. *Journal of Clinical Investigation*, *66*(5), 884-891. <https://doi.org/10.1172/jci109955>

Deng, J., Su, L.-X., Liang, Z.-X., Liang, L.-L., Yan, P., Jia, Y.-H.,…Xie, L.-X. (2013). Effects of vitamin b6 therapy for sepsis patients with linezolid-associated cytopenias: a retrospective study. *Current therapeutic research, clinical and experimental*, *74*, 26-32. <https://doi.org/10.1016/j.curtheres.2012.12.002>

Desouza, I. A., & Ribeiro-DaSilva, G. (1998). Neutrophil migration induced by staphylococcal enterotoxin type A in mice: a pharmacological analysis. *European Journal of Pharmacology*, *363*(2-3), 189-195. <https://doi.org/10.1016/s0014-2999(98)00805-x>

Dias, J., de Brito, T., Magalhaes, D., Santos, P., Batista, J., Dias, E.,…Barbosa, A. (2014). Gabapentin, a Synthetic Analogue of Gamma Aminobutyric Acid, Reverses Systemic Acute Inflammation and Oxidative Stress in Mice. *Inflammation*, *37*, 1826-1836. <https://doi.org/10.1007/s10753-014-9913-2>

Ekstrand-Hammarström, B., Österlund, C., Lilliehöök, B., & Bucht, A. (2007). Vitamin E down-modulates mitogen-activated protein kinases, nuclear factor-κB and inflammatory responses in lung epithelial cells. *Clinical and Experimental Immunology*, *147*(2), 359-369. <https://doi.org/10.1111/j.1365-2249.2006.03285.x>

Elferink, J. G. R., & Dekoster, B. M. (1991). Glutathione-Induced Enhancement of Neutrophil Locomotion. *Immunobiology*, *184*(1), 25-36. <https://doi.org/10.1016/s0171-2985(11)80569-3>

Estrela, G. R., Wasinski, F., Almeida, D. C., Amano, M. T., Castoldi, A., Dias, C. C.,…Araujo, R. C. (2014). Kinin B1 receptor deficiency attenuates cisplatin-induced acute kidney injury by modulating immune cell migration. *Journal of Molecular Medicine*, *92*(4), 399-409. <https://doi.org/10.1007/s00109-013-1116-z>

Forrester, J. V., & Lackie, J. M. (1982). Effect of Hyaluronic-Acid on Neutrophil Adhesion. *Ophthalmic Research*, *14*(5), 387-388.

Freund, H., RYAN, J., & FISCHER, J. (1978). Amino-Acid Derangements in Patients with Sepsis - Treatment with Branched-Chain Amino-Acid Rich Infusions. *Annals of Surgery*, *188*, 423-430.

Galkina, S. I., Golenkina, E. A., Fedorova, N. V., Ksenofontov, A. L., Serebryakova, M. V., Arifulin, E. A.,…Sud'ina, G. F. (2021). Inhibition of Neutrophil Secretion Upon Adhesion as a Basis for the Anti-Inflammatory Effect of the Tricyclic Antidepressant Imipramine. *Frontiers in Pharmacology*, *12*, 709719. <https://doi.org/10.3389/fphar.2021.709719>

Hong, C. Y., Huang, S. S., Wang, R., Sung, Y. J., & Kwok, C. F. (1998). Trilinolein inhibits the adhesion of neutrophils to endothelial cells. *Clinical and Experimental Pharmacology and Physiology*, *25*(2), 99-103.

Hosac, A. M. (2002). Drotrecogin alfa (activated): the first FDA-approved treatment for severe sepsis. *Proceedings (Baylor University. Medical Center)*, *15*(2), 224-227.

Ibarra-Estrada, M., Kattan, E., Aguilera-Gonzalez, P., Sandoval-Plascencia, L., Rico-Jauregui, U., Gomez-Partida, C. A.,…Hernandez, G. (2023). Early adjunctive methylene blue in patients with septic shock: a randomized controlled trial. *Critical Care*, *27*(1), 110. <https://doi.org/10.1186/s13054-023-04397-7>

Inan, M., Koyuncu, A., Aydin, C., Turan, M., Gokgoz, S., & Sen, M. (2003). Thyroid hormone supplementation in sepsis: An experimental study. *Surgery Today*, *33*, 24-29.

Jia, L., Wang, Y., Wang, Y., Ma, Y., Shen, J., Fu, Z.,…Xiang, M. (2018). Heme Oxygenase-1 in Macrophages Drives Septic Cardiac Dysfunction via Suppressing Lysosomal Degradation of Inducible Nitric Oxide Synthase. *Circulation Research*, *122*, 1532-1544. <https://doi.org/10.1161/CIRCRESAHA.118.312910>

Joshi, I., Carney, W., & Rock, E. (2023). Utility of monocyte HLA-DR and rationale for therapeutic GM-CSF in sepsis immunoparalysis. *Frontiers in Immunology*, *14*, 1130214. <https://doi.org/10.3389/fimmu.2023.1130214>

Kim, C. D., Kim, H. H., & Hong, K. W. (1999). Inhibitory effect of rebamipide on the neutrophil adherence stimulated by conditioned media from Helicobacter pylori-infected gastric epithelial cells. *Journal of Pharmacology and Experimental Therapeutics*, *288*(1), 133-138.

Kim, C. D., Kim, Y. K., Lee, S. H., & Hong, K. W. (2000). Rebamipide inhibits neutrophil adhesion to hypoxia/reoxygenation-stimulated endothelial cells via nuclear factor-κB-dependent pathway. *Journal of Pharmacology and Experimental Therapeutics*, *294*(3), 864-869.

Kim, D.-C., Lee, W., & Bae, J.-S. (2011). Vascular anti-inflammatory effects of curcumin on HMGB1-mediated responses in vitro. *Inflammation Research*, *60*(12), 1161-1168. <https://doi.org/10.1007/s00011-011-0381-y>

Krecic-Shepard, M., Shepard, D., Mullet, D., Apseloff, G., Weisbrode, S., & Gerber, N. (1999). Gallium nitrate suppresses the production of nitric oxide and liver damage in a murine model of LPS-induced septic shock. *Life Sciences*, *65*, 1359-1371.

Kumar, B., Coleman, R., & Alderson, P. (1975). Gallium Citrate GA-67 Imaging in Patients with Suspected Inflammatory Processes. *Archives of Surgery*, *110*, 1237-1242.

Lado-Abeal, J. (2020). Non-thyroidal illness syndrome, the hidden player in the septic shock induced myocardial contractile depression. *Medical Hypotheses*, *142*, 109775. <https://doi.org/10.1016/j.mehy.2020.109775>

Lee, J. H., Liu, A., Park, J.-H., Kato, H., Hao, Q., Zhang, X.,…Lee, J.-W. (2020). Therapeutic Effects of Hyaluronic Acid in Peritonitis-Induced Sepsis in Mice. *Shock*, *54*(4), 488-497. <https://doi.org/10.1097/shk.0000000000001512>

Li, X., Li, H., Wang, H., Su, L., Yan, P., Xie, L.,…Xiao, K. (2015). *Dynamic Changes in Amino Acid Concentration Profiles in Patients with Sepsis*. *PLOS One*, 10(4), doi: 10.1371/journal.pone.0121933.

Lin, S., Xiao, M., Cai, Q., Lin, Y., Yao, J., Sun, X., & Huang, J. (2023). Efficacy and Safety of Citric Acid and Heparin Anticoagulation in Patients with Septic Acute Kidney Injury Undergoing Continuous Renal Replacement Therapy: A Meta-Analysis. *Alternative Therapies in Health and Medicine*, *29*(8), 421-425.

Liu, R., Luo, X., Li, J., Lei, Y., Zeng, F., Huang, X.,…Yang, F. (2022). Melatonin: A window into the organ-protective effects of sepsis. *Biomedicine & Pharmacotherapy*, *154*, 113556. <https://doi.org/10.1016/j.biopha.2022.113556>

Lominadze, D., Saari, J. T., Percival, S. S., & Schuschke, D. A. (2004). Proinflammatory effects of copper deficiency on neutrophils and lung endothelial cells. *Immunology and Cell Biology*, *82*(3), 231-238. <https://doi.org/10.1046/j.1440-1711.2004.01231.x>

Lu, C., Leibner, E., & Wright, B. (2016). The use of tris-hydroxymethyl aminomethane in the emergency department. *Clinical and Experimental Emergency Medicine*, *3*, 264-265. <https://doi.org/10.15441/ceem.16.165>

Maayah, Z. H. H., Ferdaoussi, M., Alam, A., Takahara, S., Silver, H., Soni, S.,…Dyck, J. R. B. (2022). Cannabidiol Suppresses Cytokine Storm and Protects Against Cardiac and Renal Injury Associated with Sepsis. *Cannabis and Cannabinoid Research*. <https://doi.org/10.1089/can.2022.0170>

McHugh, D., Tanner, C., Mechoulam, R., Pertwee, R. G., & Ross, R. A. (2008). Inhibition of human neutrophil chemotaxis by endogenous cannabinoids and phytocannabinoids:: Evidence for a site distinct from CB1 and CB2. *Molecular Pharmacology*, *73*(2), 441-450. <https://doi.org/10.1124/mol.107.041863>

Miyata, R., Iwabuchi, K., Watanabe, S., Sato, N., & Nagaoka, I. (1999). Exposure of intestinal epithelial cell HT29 to bile acids and ammonia enhances Mac-1-mediated neutrophil adhesion. *Inflammation Research*, *48*(5), 265-273. <https://doi.org/10.1007/s000110050458>

Moebert, J., Zahler, S., Becker, B. F., & Conzen, P. F. (1999). Inhibition of neutrophil activation by volatile anesthetics decreases adhesion to cultured human endothelial cells. *Anesthesiology*, *90*(5), 1372-1381.

Moser, R., Groscurth, P., & Fehr, J. (1990). Promotion of Transendothelial Neutrophil Passage by Human Thrombin. *Journal of Cell Science*, *96*, 737-744.

Neeli, I., Moarefian, M., Kuseladass, J., Dwivedi, N., Jones, C., & Radic, M. (2023). Neutrophil attachment via Mac-1 (αMβ2; CD11b/CD18; CR3) integrins induces PAD4 deimination of profilin and histone H3. *Philosophical Transactions of the Royal Society B-Biological Sciences*, *378*(1890), 20220247. <https://doi.org/10.1098/rstb.2022.0247>

Newton, B., Bhat, B. V., Dhas, B. B., Mondal, N., & Gopalakrishna, S. M. (2016). Effect of Zinc Supplementation on Early Outcome of Neonatal Sepsis - A Randomized Controlled Trial. *Indian Journal of Pediatrics*, *83*(4), 289-293. <https://doi.org/10.1007/s12098-015-1939-4>

Ni, S., Yuan, Y., Kuang, Y., & Li, X. (2022). Iron Metabolism and Immune Regulation. *Frontiers in Immunology*, *13*, 816282. <https://doi.org/10.3389/fimmu.2022.816282>

Nin, N., Cassina, A., Boggia, J., Alfonso, E., Botti, H., Peluffo, G.,…Hurtado, F. J. (2004). Septic diaphragmatic dysfunction is prevented by Mn(III)porphyrin therapy and inducible nitric oxide synthase inhibition. *Intensive Care Medicine*, *30*(12), 2271-2278. <https://doi.org/10.1007/s00134-004-2427-x>

Nishio, H., Hayashi, Y., Terashima, S., & Takeuchi, K. (2008). Protective effect of pranlukast, a cysteinyl-leukotriene receptor 1 antagonist, on indomethacin-induced small intestinal damage in rats. *Inflammopharmacology*, *16*(2), 106-106. <https://doi.org/10.1007/s10787-008-0024-2>

Notcovich, S., Williamson, N., Yapura, J., Schukken, Y., & Heuer, C. (2020). Cellular Response of Neutrophils to Bismuth Subnitrate and Micronized Keratin Products In Vitro. *Veterinary Sciences*, *7*, 87. <https://doi.org/10.3390/vetsci7030087>

Nunes, P. I. G., Viana, A. F. S. C., Sasahara, G. L., Dos Santos, S. M., Alves, A. P. N. N., Silveira, E. R., & Santos, F. A. (2023). N-Methyl-(2S,4R)-trans-4-hydroxy- L-proline isolated from Sideroxylon obtusifolium attenuates TPA-induced irritant contact dermatitis in mice. *Anais Da Academia Brasileira De Ciencias*, *95*(3), e20220919. <https://doi.org/10.1590/0001-3765202320220919>

Pasini, F., Capecchi, P., Pasqui, A., Ceccatelli, L., Diperri, T., Valensin, G., & Gaggelli, E. (1990). Adenosine Blocks Calcium Entry in Activated Neutrophils and Binds to Flunarizine-Sensitive Calcium Channels. *Immunopharmacology and Immunotoxicology*, *12*, 77-91.

Peng, Z., Zhao, C., Du, X., Yang, Y., Li, Y., Song, Y.,…Liu, G. (2021). Autophagy Induced by Palmitic Acid Regulates Neutrophil Adhesion Through the Granule-Dependent Degradation of αMβ2 Integrin in Dairy Cows With Fatty Liver. *Frontiers in Immunology*, *12*, 726829. <https://doi.org/10.3389/fimmu.2021.726829>

Pennington, J. E., Kemmerich, B., Kazanjian, P. H., Marsh, J. D., & Boerth, L. W. (1986). Verapamil Impairs Human Neutrophil Chemotaxis by a Non-Calcium-Mediated Mechanism. *Journal of Laboratory and Clinical Medicine*, *108*(1), 44-52.

Protti, A., & Singer, M. (2006). Bench-to-bedside review: Potential strategies to protect or reverse mitochondrial dysfunction in sepsis-induced organ failure. *Critical Care*, *10*(5), 228. <https://doi.org/10.1186/cc5014>

Refaie, M., El-Hussieny, M., Bayoumi, A., Abdelraheem, W., Abdel-Hakeem, E., & Shehata, S. (2024). Sacubitril/valsartan alleviates sepsis-induced myocardial injury in rats via dual angiotensin receptor-neprilysin inhibition and modulation of inflammasome/caspase 1/IL1β pathway. *European Journal of Pharmacology*, *979*, G176834. <https://doi.org/10.1016/j.ejphar.2024.176834>

Sahin, S., Akoglu, T., Direskeneli, H., Sen, L. S., & Lawrence, R. (1996). Neutrophil adhesion to endothelial cells and factors affecting adhesion in patients with Behcet's disease. *Annals of the Rheumatic Diseases*, *55*(2), 128-133. <https://doi.org/10.1136/ard.55.2.128>

Sahna, E., Deniz, E., Bay-Karabulut, A., & Burma, O. (2008). Melatonin protects myocardium from ischemia-reperfusion injury in hypertensive rats: Role of myeloperoxidase activity. *Clinical and Experimental Hypertension*, *30*(7), 673-681. <https://doi.org/10.1080/10641960802251966>

Schmidt, W., Schmidt, H., Bauer, H., Gebhard, M. M., & Martin, E. (1997). Influence of lidocaine on endotoxin-induced leukocyte endothelial cell adhesion and macromolecular leakage in vivo. *Anesthesiology*, *87*(3), 617-624. <https://doi.org/10.1097/00000542-199709000-00023>

Schreckenberg, R., Wolf, A., Troidl, C., Simsekyilmaz, S., & Schlüter, K. D. (2021). Pro-inflammatory Vascular Stress in Spontaneously Hypertensive Rats Associated With High Physical Activity Cannot Be Attenuated by Aldosterone Blockade. *Frontiers in Cardiovascular Medicine*, *8*, 699283. <https://doi.org/10.3389/fcvm.2021.699283>

Shima, E., Katsube, M., Kato, T., Kitagawa, M., Hato, F., Hino, M.,…Kitagawa, S. (2008). Calcium channel blockers suppress cytokine-induced activation of human neutrophils. *American Journal of Hypertension*, *21*(1), 78-84. <https://doi.org/10.1038/ajh.2007.13>

Sikes, P. J., Zhao, P. Y., Maass, D. L., White, J., & Horton, J. W. (2005). Sodium/hydrogen exchange activity in sepsis and in sepsis complicated by previous injury:: 31P and 23Na NMR study. *Critical Care Medicine*, *33*(3), 605-615. <https://doi.org/10.1097/01.ccm.0000155910.89252.FE>

Snyder, S. L., & Walker, R. I. (1976). Inhibition of Lethality in Endotoxin-Challenged Mice Treated with Zinc CHloride. *Infection and Immunity*, *13*(3), 998-1000. <https://doi.org/10.1128/iai.13.3.998-1000.1976>

Soylu, S., & Inan, Z. (2018). Effect of Strontium Ranelate on Multiple Organ Damage in a Rat Sepsis Model. *Iranian Red Crescent Medical Journal*, *20*, e68840. <https://doi.org/10.5812/ircmj.68840>

Takeuchi, K., Ito, K., & Namikawa, S. (1988). Anti-Inflammatory Activity of the Dry Distillation Tar of Delpidated Soybeen (Glyteer) .2. *Folia Pharmacologica Japonica*, *91*(1), 1-7. <https://doi.org/10.1254/fpj.91.1>

Toyosawa, T., Suzuki, M., Kodama, K., & Araki, S. (2004). Highly purified vitamin B2 presents a promising therapeutic strategy for sepsis and septic shock. *Infection and Immunity*, *72*(3), 1820-1823. <https://doi.org/10.1128/iai.72.3.1820-1823.2004>

Tuon, L., Comim, C. M., Antunes, M. M., Constantino, L. S., Machado, R., Izquierdo, I.,…Dal-Pizzol, F. (2007). Imipramine reverses the depressive symptoms in sepsis survivor rats. *Intensive Care Medicine*, *33*(12), 2165-2167. <https://doi.org/10.1007/s00134-007-0804-y>

Unoshima, M., Nishizono, A., Takita-Sonoda, Y., Iwasaka, H., & Noguchi, T. (2001). Effects of zinc acetate on splenocytes of endotoxemic mice: Enhanced immune response, reduced apoptosis, and increased expression of heat shock protein 70. *Journal of Laboratory and Clinical Medicine*, *137*(1), 28-37. <https://doi.org/10.1067/mlc.2001.111514>

Vadas, P., Stefanski, E., & Pruzanski, W. (1986). Potential Therapeutic Efficacy of Inhibitors of Human Phospholipase-A2 in Septic Shock. *Agents and Actions*, *19*(3-4), 194-202. <https://doi.org/10.1007/bf01966206>

Verdrengh, M., & Tarkowski, A. (2005). Riboflavin in innate and acquired immune responses. *Inflammation Research*, *54*(9), 390-393. <https://doi.org/10.1007/s00011-005-1372-7>

Vlahakos, D., Arkadopoulos, N., Kostopanagiotou, G., Siasiakou, S., Kaklamanis, L., Degiannis, D.,…Smyrniotis, V. (2012). Deferoxamine Attenuates Lipid Peroxidation, Blocks Interleukin-6 Production, Ameliorates Sepsis Inflammatory Response Syndrome, and Confers Renoprotection After Acute Hepatic Ischemia in Pigs. *Artificial Organs*, *36*(4), 400-408. <https://doi.org/10.1111/j.1525-1594.2011.01385.x>

Walter, T., Arredondo, S., Arevalo, M., & Stekel, A. (1986). Effect of Iron Therapy on Phagocytosis and Bactericidal Activity in Neutrophils of Iron-Deficient Infants. *American Journal of Clinical Nutrition*, *44*, 877-882.

Wang, D., Zheng, J., Hu, Q., Zhao, C., Chen, Q., Shi, P.,…Lin, Z. (2020). Magnesium protects against sepsis by blocking gasdermin D N-terminal-induced pyroptosis. *Cell Death and Differentiation*, *27*, 466-481. <https://doi.org/10.1038/s41418-019-0366-x>

Wang, J., Li, J., Lou, A., Lin, Y., Xu, Q., Cui, W.,…Li, X. (2023). Sacubitril/valsartan alleviates sepsis-induced acute lung injury via inhibiting GSDMD-dependent macrophage pyroptosis in mice. *Febs Journal*, *290*(8), 2180-2198. <https://doi.org/10.1111/febs.16696>

Wang, R., Zhu, Y., Liu, Z., Chang, L., Bai, X., Kang, L.,…Zhao, B. (2021). Neutrophil extracellular traps promote tPA-induced brain hemorrhage via cGAS in mice with stroke. *Blood*, *138*, 91-103. <https://doi.org/10.1182/blood.2020008913>

Xia, Y., Farah, N., Maxan, A., Zhou, J., & Lehmann, C. (2016). Therapeutic iron restriction in sepsis. *Medical Hypotheses*, *89*, 37-39. <https://doi.org/10.1016/j.mehy.2016.01.018>

Xu, Y., Xue, Y., Liu, X., Li, Y., Liang, H., Dou, H., & Hou, Y. (2019). Ferumoxytol Attenuates the Function of MDSCs to Ameliorate LPS-Induced Immunosuppression in Sepsis. *Nanoscale Research Letters*, *14*, 379. <https://doi.org/10.1186/s11671-019-3209-2>

Yang, J., Zhang, R., Zhao, H., Qi, H., Li, J., Li, J.-F.,…Zhang, T. (2022). Bioinspired copper single-atom nanozyme as a superoxide dismutase-like antioxidant for sepsis treatment. *Exploration*, *2*(4), 20210267-20210267. <https://doi.org/10.1002/exp.20210267>

Ye, M., Zhao, Y., Wang, Y., Xie, R., Tong, Y., Sauer, J.-D., & Gong, S. (2022). NAD(H)-loaded nanoparticles for efficient sepsis therapy via modulating immune and vascular homeostasis. *Nature Nanotechnology*, *17*(8), 880-+. <https://doi.org/10.1038/s41565-022-01137-w>
