# Supplementary material for: Prioritizing FDA approved therapeutics for treating sepsis phenotypes: A network modeling approach based on neutrophil proteomics
Source: Front Immunol. 2025 Aug 14;16:1646141. doi: 10.3389/fimmu.2025.1646141 (PMC12391923; doi:10.3389/fimmu.2025.1646141)
Supplement: Supplementary file 4 [file Table4.docx]

**Summary statistics for STRING network**

**
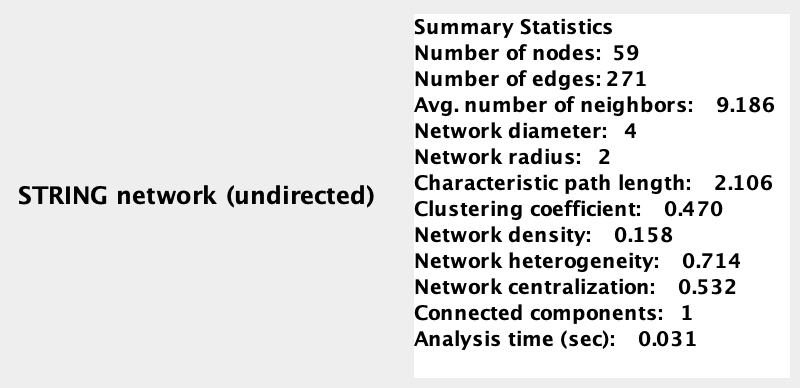
**

Summary network statistics from Cytoscape on the STRING network of DEPs targeted by FDA-approved therapeutics or (pre)clinical trial therapeutics.

**Explanation of summary statistics**

Number of nodes: This value represents the total number of biological entities (i.e., proteins) in the network.

Number of edges: This value represents the total number of interactions between nodes in the network.

Average number of neighbors: This value indicates the average connectivity of a node in the network.

Network diameter: This value represents the largest distance between two nodes.

Network radius: This value represents half the diameter

Characteristic path length: This value gives the expected distance between two connected nodes.

Clustering coefficient: This value is the ratio N / M, where N is the number of edges between the neighbors of n and M is the maximum number of edges that could potentially exist between the neighbors of n. The value is always between 0 and 1.

Network density: This is a normalized version of the average number of neighbors statistic, and it has a value between 0 and 1. It indicates how densely the network is populated with edges (excluding duplicated edges and self loops).

Network heterogeneity: This value shows the tendency of a network to contain hub nodes.

Network centralization: This parameter measures how connected a group of nodes are populated around a single node or a group of nodes; in other words, it measures the extent by which a network is centered around a node.

Connected components: If a set of nodes, in an undirected graph, are all connected via a set of edges so that each node is reachable by traversing edges, then the set of nodes for a connected component.

Analysis time: The duration of time for Cytoscape to analyze the network.

**More information on these network statistical parameters can be found elsewhere:**

https://bookdown.org/markhoff/social_network_analysis/centrality.html

**CytoHubba results**

| Rank | Name | Score |
| --- | --- | --- |
| 1 | ALB | 39 |
| 2 | APP | 28 |
| 3 | CDC42 | 25 |
| 4 | CD74 | 19 |
| 5 | TTR | 18 |
| 6 | PRTN3 | 15 |
| 6 | VTN | 15 |
| 8 | TSPO | 14 |
| 9 | PDHA1 | 13 |
| 10 | PPP2CA | 12 |
| 10 | ATP2B1 | 12 |
| 10 | FTH1 | 12 |
| 10 | MIF | 12 |
| 10 | PPT1 | 12 |
| 10 | TLR1 | 12 |
| 10 | TF | 12 |
| 17 | GNB1 | 11 |
| 17 | IDH3A | 11 |
| 17 | CYB5R3 | 11 |
| 17 | FPR1 | 11 |
| 21 | PAK1 | 10 |
| 21 | RALA | 10 |
| 21 | FLNC | 10 |
| 21 | BLVRB | 10 |
| 25 | FGG | 9 |
| 25 | NDUFV2 | 9 |
| 25 | ALPL | 9 |
| 25 | DDX3Y | 9 |
| 29 | ORM2 | 8 |
| 29 | PMPCA | 8 |
| 31 | DHCR7 | 7 |
| 31 | TPP1 | 7 |
| 31 | PRG2 | 7 |
| 31 | NDUFA2 | 7 |
| 31 | PDP1 | 7 |
| 31 | HIBADH | 7 |
| 31 | BAZ1B | 7 |
| 31 | MME | 7 |
| 39 | TRPV2 | 6 |
| 39 | RNASE3 | 6 |
| 39 | UGCG | 6 |
| 42 | HIST2H2AB | 5 |
| 42 | CACNA1G | 5 |
| 42 | EED | 5 |
| 42 | GSTM2 | 5 |
| 46 | TAOK1 | 4 |
| 46 | ALG1 | 4 |
| 46 | EXOC8 | 4 |
| 46 | HAPLN1 | 4 |
| 46 | EPX | 4 |
| 46 | SP110 | 4 |
| 52 | PPIH | 3 |
| 52 | MIA3 | 3 |
| 52 | UBE4A | 3 |
| 52 | SPPL2A | 3 |
| 56 | LRSAM1 | 2 |
| 56 | MBOAT7 | 2 |
| 58 | DAGLB | 1 |
| 58 | ASRGL1 | 1 |

**Hubs**

| **Functional phenotype** | **Fold change in protein expression as compared to control** | **Hub** | **Degree (number of connections with other proteins)** |
| --- | --- | --- | --- |
| Hybrid | 0.3 | ALB | 39 |
| Hybrid | 0.25 | APP | 28 |
| Hyperimmune | 0.17 | CDC42 | 25 |
| Hyperimmune, Hypoimmune, Hybrid | (45, 8, 8) | CD74 | 19 |
| Hyperimmune | -0.13 | TTR | 18 |
| Hyperimmune | 4.9 | PRTN3 | 15 |
| Hybrid | 11 | VTN | 15 |
| (Hyperimmune, Hypoimmune) | (100, 100) | TSPO | 14 |
| (Hyperimmune, Hybrid) | (9, 6) | PDHA1 | 13 |
| Hybrid | 0.01 | ATP2B1 | 12 |
| (Hyperimmune, Hypoimmune, Hybrid) | (5, 2, 6) | FTH1 | 12 |
| (Hyperimmune, Hybrid) | (6, 5) | MIF | 12 |
| (Hyperimmune, Hypoimmune, Hybrid) | (.04, .14, .02) | PPP2CA | 12 |
| Hyperimmune | 5 | PPT1 | 12 |
| Hybrid | 0.29 | TF | 12 |
| (Hyperimmune, Hybrid) | (100, 100) | TLR1 | 12 |
| Hypoimmune | 2 | CYB5R3 | 11 |
| (Hypoimmune, Hybrid) | (100, 100) | FPR1 | 11 |
| (Hyperimmune, Hybrid) | (.03, .04) | GNB1 | 11 |
| Hybrid | 5 | IDH3A | 11 |
| Hypoimmune | 0.34 | BLVRB | 10 |
| Hybrid | 0.32 | FLNC | 10 |
| Hybrid | 0.27 | PAK1 | 10 |
| Hypoimmune | 4 | RALA | 10 |
| (Hyperimmune, Hybrid) | (6, 8) | ALPL | 9 |
| Hybrid | 0.09 | DDX3Y | 9 |
| (Hyperimmune, Hybrid) | (2, 8) | FGG | 9 |
| Hypoimmune | 5 | NDUFV2 | 9 |
| Hybrid | 0.21 | ORM2 | 8 |
| Hypoimmune | 0.01 | PMPCA | 8 |
| Hypoimmune | 0.32 | BAZ1B | 7 |
| Hypoimmune | 0.28 | DHCR7 | 7 |
| Hypoimmune | 0.01 | HIBADH | 7 |
| Hybrid | 0.15 | MME | 7 |
| Hypoimmune | 0.21 | NDUFA2 | 7 |
| Hypoimmune | 0.01 | PDP1 | 7 |
| (Hyperimmune, Hybrid) | (.05, .11) | PRG2 | 7 |
| (Hyperimmune, Hybrid) | (9, 7) | TPP1 | 7 |
| Hypoimmune | 0.39 | RNASE3 | 6 |
| Hypoimmune | 6 | TRPV2 | 6 |
| (Hyperimmune, Hybrid) | (.20, .24) | UGCG | 6 |
| (Hyperimmune, Hybrid) | (.01, .03) | CACNA1G | 5 |
| (Hyperimmune, Hypoimmune, Hybrid) | (100, 100, 100) | EED | 5 |
| (Hyperimmune, Hybrid) | (.16, .23) | GSTM2 | 5 |
| Hyperimmune | 3 | H2AC21 | 5 |
